# Prognostic Significance of Oxidative Stress Biomarkers and Novel Hematological Inflammatory Indices in Colorectal Cancer: A Prospective Observational Study

**DOI:** 10.3390/medicina62091646

**Published:** 2026-08-27

**Authors:** Răzvan Alexandru Marinescu, Daniela Marinescu, Ana-Maria Ciurea, Lidia Boldeanu, Marius Bică, Ștefan Pătrașcu, Victor Dan Eugen Strâmbu, Petru Adrian Radu, Petrica Popa, Mihail Virgil Boldeanu, Styliani Laskou, Valeriu Șurlin

**Affiliations:** 1 Doctoral School, University of Medicine and Pharmacy of Craiova, 200349 Craiova, Romania; razvanalexandrumarinescu98@gmail.com; 2 Department of Surgery, University of Medicine and Pharmacy of Craiova, 200349 Craiova, Romania; daniela.marinescu@umfcv.ro (D.M.); marius.bica@umfcv.ro (M.B.); stefan.patrascu@umfcv.ro (Ș.P.); vsurlin@gmail.com (V.Ș.); 3 Department of Oncology, University of Medicine and Pharmacy of Craiova, 200349 Craiova, Romania; 4 Department of Microbiology, Faculty of Medicine, University of Medicine and Pharmacy of Craiova, 200349 Craiova, Romania; 5 Department of Surgery, University of Medicine and Pharmacy “Carol Davila”, 020021 Bucharest, Romania; victor.strambu@umfcd.ro (V.D.E.S.); petru.radu@umfcd.ro (P.A.R.); 6 Department of Gastroenterology, University of Medicine and Pharmacy of Craiova, 200349 Craiova, Romania; petrica.popa@umfcv.ro; 7 Department of Immunology, Faculty of Medicine, University of Medicine and Pharmacy of Craiova, 200349 Craiova, Romania; mihail.boldeanu@umfcv.ro; 8 3rd Department of Surgery, School of Medicine, Aristotle University of Thessaloniki, “AHEPA” University Hospital, 54621 Thessaloniki, Greece; stelaskou@gmail.com

**Keywords:** colorectal cancer, oxidative stress, malondialdehyde, 8-epi-prostaglandin F2α, superoxide dismutase 1, inflammatory biomarkers, mean corpuscular volume-to-lymphocyte ratio (MCVL), cumulative inflammatory index (IIC), prognosis, survival analysis, lipid peroxidation, systemic inflammation

## Abstract

*Background and Objectives*: Oxidative stress and chronic inflammation are closely interconnected processes involved in colorectal cancer (CRC) development and progression. However, the relationships between oxidative stress biomarkers, emerging hematological inflammatory indices, clinicopathological characteristics, and survival outcomes remain insufficiently characterized. This study evaluated circulating levels of 8-epi-prostaglandin F2α (8-epi-PGF2α), malondialdehyde (MDA), and superoxide dismutase 1 (SOD1), together with conventional and novel inflammatory indices, including the mean corpuscular volume-to-lymphocyte ratio (MCVL) and cumulative inflammatory index (IIC), in patients with CRC. *Materials and Methods*: This prospective observational study included 140 patients with histologically confirmed CRC and 40 healthy controls. Serum concentrations of 8-epi-PGF2α, MDA, and SOD1 were measured by ELISA, and hematological inflammatory indices were calculated from complete blood counts. Associations with clinicopathological characteristics were evaluated using non-parametric analyses with correction for multiple testing where appropriate. Spearman correlations with false discovery rate correction were used to assess oxidative–inflammatory associations. Prognostic analyses included 24-month time-dependent receiver operating characteristic (ROC) analysis accounting for censoring, Kaplan–Meier analysis, and Cox proportional hazards regression for overall survival (OS) and progression-free survival (PFS). *Results*: CRC patients exhibited significantly higher serum levels of 8-epi-PGF2α (*p* = 0.016), MDA (*p* < 0.001), and SOD1 (*p* < 0.001) than controls. Oxidative stress biomarkers differed significantly across TNM stage, nodal status, and histological grade, although the observed patterns were not uniformly progressive with disease stage. After false discovery rate correction, MDA retained significant correlations with multiple hematological inflammatory parameters, whereas SOD1 showed a more restricted correlation profile and 8-epi-PGF2α showed no significant correlations. At 24 months, MDA demonstrated the highest time-dependent discrimination for OS (AUC = 0.920; bootstrap 95% CI: 0.834–0.978), whereas its discrimination for PFS was modest (AUC = 0.636; bootstrap 95% CI: 0.487–0.773). High MDA, defined by the internally derived 24-month threshold, was associated with shorter PFS after adjustment for age, sex, and TNM stage (HR = 2.49, 95% CI: 1.29–4.80; *p* = 0.006) and, separately, metastatic status (HR = 2.40, 95% CI: 1.22–4.72; *p* = 0.011). However, when modeled continuously, MDA was no longer significantly associated with PFS after multivariable adjustment. *Conclusions*: CRC was associated with increased circulating oxidative stress biomarkers, with MDA showing the most consistent relationships with systemic inflammatory parameters and survival outcomes. Its prognostic association with PFS was dependent on the modeling approach, while its high discrimination for 24-month OS should be interpreted cautiously because of the limited number and metastatic restriction of death events. These findings identify MDA as a promising candidate oxidative–inflammatory marker warranting external validation rather than an independently established prognostic biomarker.

## 1. Introduction

Colorectal cancer (CRC) remains a major global health burden and one of the leading causes of cancer-related morbidity and mortality worldwide, representing the third most frequently diagnosed malignancy and the second leading cause of cancer-associated death globally [1]. Despite substantial advances in screening, surgical techniques, molecular profiling, and systemic therapies, prognosis remains strongly dependent on disease stage, with recurrence, metastatic progression, and treatment resistance continuing to account for a substantial proportion of CRC-related mortality. This clinical heterogeneity underscores the need for accessible biomarkers that complement conventional clinicopathological staging and improve prognostic stratification. The biological mechanisms underlying colorectal carcinogenesis are complex and involve genomic instability, chronic inflammation, metabolic dysregulation, immune alterations, and oxidative stress [2,3].

Oxidative stress is a state of imbalance between excessive production of reactive oxygen species (ROS) and the antioxidant defense mechanisms that neutralize them. Persistent ROS accumulation induces oxidative damage to cellular lipids, proteins, and nucleic acids, thereby contributing to mutagenesis, abnormal intracellular signaling, mitochondrial dysfunction, and malignant transformation [4,5]. Increasing evidence suggests that oxidative stress plays a critical role not only in CRC initiation but also in tumor progression, angiogenesis, invasion, and metastatic dissemination [2,6].

Among the most important consequences of oxidative stress is lipid peroxidation, which generates highly reactive metabolites that can amplify carcinogenic processes. In the present study, 8-epi-prostaglandin F2α (8-epi-PGF2α), malondialdehyde (MDA), and superoxide dismutase 1 (SOD1) were selected because they reflect complementary components of the oxidative balance. MDA is one of the most extensively investigated end-products of lipid peroxidation and has been associated with tumor aggressiveness, metastatic potential, and unfavorable oncological outcomes in gastrointestinal malignancies [7]. Similarly, 8-epi-PGF2α, a stable F2-isoprostane generated through free-radical-mediated peroxidation of arachidonic acid, is considered a sensitive biomarker of in vivo lipid peroxidation and systemic oxidative injury [8].

In contrast to these markers of oxidative lipid damage, SOD1 reflects endogenous antioxidant defense. This enzyme catalyzes the dismutation of superoxide radicals into hydrogen peroxide and molecular oxygen, and it is a major component of the cellular response to oxidative stress. Although antioxidant systems physiologically protect tissues against oxidative injury, sustained activation of antioxidant pathways in cancer may paradoxically facilitate tumor adaptation to oxidative stress, resistance to apoptosis, and metastatic progression [5,9]. Thus, the combined assessment of 8-epi-PGF2α and MDA as indicators of oxidative injury and SOD1 as an indicator of antioxidant defense was intended to characterize complementary aspects of redox homeostasis rather than a single component of oxidative stress.

Recent studies have emphasized the close interaction between oxidative stress and systemic inflammation in CRC. Reactive oxygen species can activate multiple pro-inflammatory and oncogenic pathways, including NF-κB, STAT3, PI3K/Akt, and HIF-1α signaling, thereby promoting angiogenesis, epithelial–mesenchymal transition, immune dysregulation, and tumor dissemination [4,10]. In parallel, systemic inflammatory activation contributes to additional ROS generation, creating a self-perpetuating oxidative–inflammatory microenvironment that supports tumor progression [3,11,12,13]. Persistent activation of this bidirectional oxidative–inflammatory axis may further facilitate tumor cell survival, invasion, metastatic dissemination, and resistance to anticancer therapy, providing a biological rationale for the association of circulating oxidative stress and inflammatory biomarkers with disease progression and adverse clinical outcomes [2,3,12,13].

In this context, hematological inflammatory indices derived from routine blood parameters have emerged as promising prognostic biomarkers in oncology. Ratios such as neutrophil-to-lymphocyte ratio (NLR), platelet-to-lymphocyte ratio (PLR), monocyte-to-lymphocyte ratio (MLR), systemic immune-inflammation index (SII), systemic inflammation response index (SIRI), and aggregate index of systemic inflammation (AISI) have demonstrated prognostic relevance in CRC by reflecting the balance between tumor-promoting inflammation and antitumor immune response. In addition to established indices such as NLR, PLR, MLR, SII, SIRI, and AISI, we included two less extensively investigated hematological indices, the mean corpuscular volume-to-lymphocyte ratio (MCVL) and cumulative inflammatory index (IIC), to explore whether they provide complementary information on the systemic inflammatory profile of CRC. MCVL integrates mean corpuscular volume with lymphocyte count and may therefore reflect the relationship between erythrocyte-related alterations and host immune status. IIC integrates erythrocyte-related parameters (MCV and RDW) with neutrophil and lymphocyte counts into a composite index intended to capture complementary hematological and inflammatory information. Because evidence regarding these indices in CRC remains limited, their inclusion in the present study was exploratory and aimed at assessing their relationships with oxidative stress biomarkers and clinicopathological characteristics rather than establishing them as validated prognostic markers [14].

Although oxidative stress biomarkers and hematological inflammatory indices have been individually associated with CRC progression and prognosis, these two biological dimensions have largely been investigated separately. Consequently, the relationship between circulating markers of lipid peroxidation and antioxidant defense and established or emerging composite inflammatory indices remains insufficiently characterized. In particular, limited evidence is available on whether oxidative stress biomarkers such as 8-epi-PGF2α, MDA, and SOD1 are associated with newer indices such as MCVL and IIC, and whether these relationships vary with clinicopathological disease characteristics [2,13]. Moreover, the simultaneous evaluation of these oxidative and inflammatory markers in relation to tumor stage, metastatic status, histological characteristics, and survival outcomes has not been extensively explored.

Therefore, the present study aimed to characterize the systemic oxidative stress profile in patients with CRC by simultaneously assessing serum levels of 8-epi-PGF2α, MDA, and SOD1, and to investigate their relationships with conventional and emerging hematological inflammatory indices, including MCVL and IIC. We further evaluated these biomarkers in relation to clinicopathological characteristics and survival outcomes, including overall survival (OS) and progression-free survival (PFS). By integrating oxidative stress, systemic inflammation, tumor characteristics, and longitudinal outcomes within a single cohort, the study sought to determine whether these complementary biological measures provide additional information for CRC progression and prognostic stratification.

## 2. Materials and Methods

### 2.1. Study Design and Patient Selection

This prospective, observational, single-center cohort study was conducted at the 1st Clinic of Surgery of the Emergency County Clinical Hospital of Craiova, Romania, between January 2024 and January 2026. During the study period, consecutive patients with newly diagnosed, histologically confirmed CRC who were admitted to the surgical department were screened for eligibility. Patients who met the predefined inclusion and exclusion criteria were invited to participate and enrolled after providing written informed consent. A total of 140 consecutive patients with CRC were included in the study. The diagnosis of CRC was established based on clinical, endoscopic, imaging, and histopathological evaluations. The reporting of this observational study followed the Strengthening the Reporting of Observational Studies in Epidemiology (STROBE) recommendations.

A control group consisting of 40 healthy subjects, frequency-matched to the CRC cohort by age and sex at the group level, without known malignant, inflammatory, infectious, or autoimmune diseases was also included. Control subjects underwent routine clinical and laboratory evaluation before enrollment.

### 2.2. Inclusion and Exclusion Criteria

CRC patients. Patients were eligible for inclusion if they had histologically confirmed colorectal cancer and fulfilled the predefined study eligibility requirements. Exclusion criteria were previous oncological treatment before blood sampling; active inflammatory, infectious, or autoimmune diseases; severe hepatic or renal dysfunction; and other conditions specified in the study protocol that could substantially influence systemic inflammatory or oxidative stress parameters.

Healthy controls were recruited during the same study period from the outpatient Gastroenterology and Surgery services of the same hospital and consisted of individuals presenting for routine clinical investigations. Controls were frequency-matched to the CRC cohort by age and sex at the group level; no individual case-to-control matching was performed. Individuals with a known history of malignancy or with active inflammatory, infectious, or autoimmune diseases were excluded. A total of 75 potential healthy controls were assessed for eligibility; 35 were excluded for inflammatory, infectious, or autoimmune diseases, resulting in a final control group of 40 participants.

**Figure 1:**
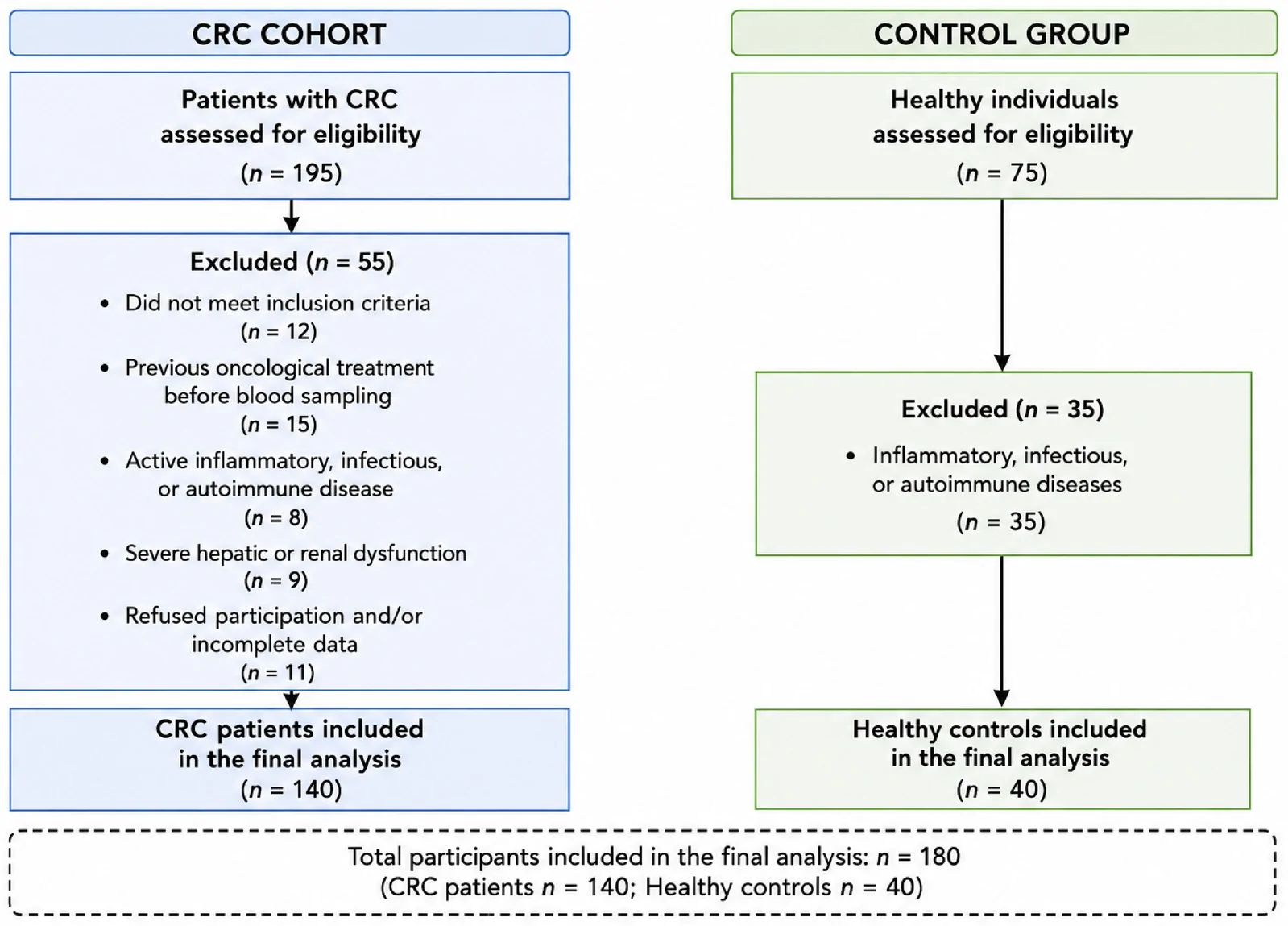
Flowchart of participant recruitment, eligibility assessment, exclusions, and final inclusion in the study. CRC: colorectal cancer.

During the recruitment period, 195 patients with CRC were assessed for eligibility. Of these, 55 were excluded because they did not meet the inclusion criteria (*n* = 12); had received prior oncological treatment before blood sampling (*n* = 15); had active inflammatory, infectious, or autoimmune diseases (*n* = 8); had severe hepatic or renal dysfunction (*n* = 9); or refused participation and/or had incomplete data (*n* = 11). Consequently, 140 patients with CRC were included in the final analysis. The participant recruitment and inclusion process is summarized in Figure 1.

No formal a priori sample size calculation was performed. The study used a consecutive sampling approach, and all eligible patients with CRC who met the inclusion criteria during the predefined recruitment period were invited to participate. Therefore, the final sample size reflected the number of eligible participants available during the study period. The control group was recruited during the same study period using predefined eligibility criteria.

The study protocol was approved by the Ethics Committee of the University of Medicine and Pharmacy of Craiova (approval no. 235/4 October 2023), and all participants provided written informed consent prior to inclusion.

Information regarding adjuvant chemotherapy and radiotherapy was recorded for all patients. Detailed information regarding surgical procedures, neoadjuvant therapy, and systemic treatment for metastatic disease was not available in a standardized manner.

### 2.3. Clinical and Histopathological Assessment

Clinical data, including age, sex, area of residence, tumor location, tumor grade, and TNM stage, were collected from medical records and pathology reports. Tumor staging was performed according to the TNM Classification of Malignant Tumors and WHO Classification of Tumors (2019) [14]. Tumor extension was classified as T1–T4, regional lymph node involvement as N0–N2, and distant metastasis as M0–M1. Histological grading was categorized into G1 (well differentiated), G2 (moderately differentiated), and G3 (poorly differentiated) tumors.

### 2.4. Laboratory Investigations

Sample Collection

Following confirmation of the CRC diagnosis, peripheral blood samples were collected before the initiation of any oncological treatment, including surgery, chemotherapy, radiotherapy, or other systemic anticancer therapy. Therefore, all oxidative stress biomarkers, hematological parameters, and derived inflammatory indices analyzed in this study were determined from pretreatment blood samples. During the biological sampling process, blood samples were collected into separate tubes according to the subsequent laboratory analyses, as follows:

• One additive-free tube (Becton Dickinson Vacutainer, Franklin Lakes, NJ, USA) was used to collect about 5 mL of venous blood from each patient. Following collection, blood samples intended for serum separation were allowed to clot and were centrifuged at 3000× *g* for 10 min using a Hermle centrifuge (Hermle Labortechnik GmbH, Wehingen, Germany) within 4 h of collection. All samples were processed according to the same standardized protocol. After centrifugation, serum was immediately separated, aliquoted into sterile polypropylene tubes, and stored at −80 °C in a certified medical ultra-low-temperature freezer until biomarker analysis. Aliquoting was performed to minimize repeated freeze–thaw cycles, and samples were thawed only when required for ELISA measurements. Before analysis, the frozen serum was gently thawed to room temperature. These aliquots were used for immunological testing, while the serum from the second tube was kept for biochemical analyses.
• Peripheral venous blood collected in EDTA Vacutainer tubes (Becton Dickinson Vacutainer, Franklin Lakes, NJ, USA) was used to perform a complete blood count (CBC). Using flow cytometry principles, we developed an extended leukocyte differential by analyzing five parameters on the automatic hematology analyzer (Alinity, Abbott, Abbott Park, IL, USA). This method enabled us to accurately identify and characterize various hematological markers: hemoglobin (Hb), white blood cells/leukocytes (WBC), neutrophils (NEU), lymphocytes (LYM), monocytes (MON), platelets (PLT), and hematocrit (Ht). For calculation of the hematological inflammatory indices, absolute NEU, LYM, MON, and PLT counts were expressed in ×10^3^/µL, MCV in fL, and RDW in %. The same units were used consistently for all patients and controls. The indices were calculated as follows:
  − NLR = neutrophil-to-lymphocyte ratio;
  − MLR = monocyte-to-lymphocyte ratio;
  − PLR = platelet-to-lymphocyte ratio;
  − AISI = (neutrophils × monocytes × platelets)/lymphocytes;
  − SII = (neutrophils × platelets)/lymphocytes;
  − SIRI = (neutrophils × monocytes)/lymphocytes;
  − MCVL = mean corpuscular volume to lymphocyte ratio;
  − IIC = (mean corpuscular volume × width of erythrocyte distribution × neutrophils)/(lymphocytes × 1000) [13,15,16,17,18,19]. For MCVL and IIC calculations, lymphocyte and neutrophil values were entered as absolute counts expressed in ×10^3^/µL. The factor of 1000 in the IIC formula was retained in accordance with the previously published definition of this index [13,15,16,17,18,19].

### 2.5. Immunological Assessment

Serum levels of 8-epi-PGF2α, SOD1, and MDA were measured using the Enzyme-Linked Immunosorbent Assay (ELISA) in the Immunology Laboratory at the University of Medicine and Pharmacy of Craiova.

We utilized commercially available test kits from Elabscience (Houston, TX, USA), each tailored for the specific mediators listed below:

• 8-epi-PGF2α (8-Epi-Prostaglandin F2 Alpha) ELISA Kit [Catalog No.: E-EL-0041; Product Link: https://www.elabscience.com/p/8-epi-pgf2-8-epi-prostaglandin-f2-alpha-elisa-kit--e-el-0041 (accessed on 25 April 2026); Sensitivity: 9.38 pg/mL; Detection range: 15.63–1000 pg/mL; Specificity: No significant cross-reactivity or interference be-tween 8 epi-PGF2α and analogs was observed; Intra-/Inter-Assay CV (%): Coefficient of variation is <10%; https://789.bio/ea/OC4Om5; (accessed on 25 April 2026)];
• Human MDA(Malondialdehyde) ELISA Kit [Catalog No.: E-EL-0060; Product Link: https://www.elabscience.com/p/mda-malondialdehyde-elisa-kit--e-el-0060 (accessed on 25 April 2026); Sensitivity: 18.75 ng/mL; Detection range: 31.25–2000 ng/mL; Specificity: No significant cross-reactivity or interference be-tween Human MDA and analogs was observed; Intra-/Inter-Assay CV (%): Coefficient of variation is <10%; https://789.bio/ea/GG8Gy9; (accessed on 25 April 2026)];
• Human SOD1 (Superoxide Dismutase 1, Soluble) ELISA Kit [Catalog No.: E-EL-H1113; Product Link: https://www.elabscience.com/p/human-sod1-superoxide-dismutase-1-soluble-elisa-kit--e-el-h1113 (accessed on 25 April 2026); Sensitivity: 37.5 pg/mL; Detection range: 62.5–4000 pg/mL; Specificity: No significant cross-reactivity or interference between Human SOD1 and analogs was observed; Intra-/Inter-Assay CV (%): Coefficient of variation is <10%; https://789.bio/ea/vnXzfH; (accessed on 25 April 2026)].

Each serum sample was analyzed once using the standardized laboratory protocol. Because of the available sample volume and study resources, duplicate measurements were not performed. Although intra- and inter-assay coefficients of variation were not independently assessed in this study, the analytical performance characteristics of the assays were based on the manufacturer’s validation data. After thawing, each sample was diluted according to the manufacturer’s instructions or following the laboratory’s internal working protocol.

Summary of test principle: This assay is based on the sandwich ELISA technique. Human 8-epi-PGF2α, SOD1, and MDA present in the sample are specifically bound by immobilized capture antibodies and subsequently recognized by biotinylated detection antibodies, forming sandwich immune complexes. This complex is further associated with an avidin–horseradish peroxidase (HRP) conjugate, enabling enzymatic signal amplification. Upon reaction with a chromogenic substrate, the HRP enzyme catalyzes the development of a colored product. The resulting signal intensity, measured spectrophotometrically at 450 ± 2 nm, is directly proportional to the concentration of human 8-epi-PGF2α, SOD1, and MDA in the sample. Quantitative determination is achieved by comparison with a standard curve generated from known concentrations.

### 2.6. Statistical Analysis

Statistical analyses were performed using GraphPad Prism version 11.0.2 (92) (GraphPad Software LLC, San Diego, CA, USA). The distribution of continuous variables was assessed using the Shapiro–Wilk normality test. Continuous variables with an approximately normal distribution were summarized as mean ± standard deviation (SD), whereas non-normally distributed continuous variables were presented as median and interquartile range (IQR; 25th–75th percentiles). Categorical variables were reported as absolute frequencies and percentages.

Participants with incomplete data required for the predefined analyses were excluded during the eligibility and data-quality assessment, as shown in the participant flowchart. Among the 140 CRC patients and 40 healthy controls included in the final analytical cohort, no missing observations were present for the variables used in the reported analyses. Therefore, no data imputation was performed, and all analyses were conducted using complete observations.

For group comparisons, differences between CRC patients and healthy controls were assessed using the Mann–Whitney U test for non-normally distributed continuous variables. For the primary comparisons of oxidative stress biomarkers between CRC patients and healthy controls, rank-biserial correlations were calculated as effect-size estimates for the Mann–Whitney U tests, with corresponding 95% confidence intervals obtained by bootstrap resampling. Differences among more than two independent groups, including TNM stages, tumor invasion (T stage), nodal status (N stage), metastatic status (M stage), and histological grade (G1–G3), were evaluated using the Kruskal–Wallis test followed by Dunn’s multiple-comparison post hoc test with Bonferroni adjustment when overall significance was detected. Comparisons according to tumor sidedness (right- versus left-sided tumors) were performed using the Mann–Whitney U test. Given the multiplicity of the secondary and exploratory clinicopathological subgroup analyses, the Benjamini–Hochberg procedure was additionally applied within each predefined family of related tests to control the false discovery rate (FDR).

Associations between oxidative stress biomarkers and hematological parameters or inflammatory indices were assessed using Spearman’s rank correlation analysis. Correlation coefficients (*ρ*) were interpreted according to conventional criteria, with values of 0.10–0.29 considered weak, 0.30–0.49 moderate, and ≥0.50 strong correlations. Given the number of correlation tests performed, the Benjamini–Hochberg procedure was applied to control the FDR across the correlation analyses. FDR-adjusted *p*-values (*q*-values) were calculated, with *q* < 0.05 considered statistically significant.

Time-dependent receiver operating characteristic (ROC) curve analyses were performed to evaluate the discriminatory performance of oxidative stress biomarkers for overall survival (OS) and progression-free survival (PFS) at a clinically relevant 24-month prediction horizon. A cumulative/dynamic time-dependent ROC approach using inverse probability of censoring weighting (IPCW) was applied to account for censored observations. The time-dependent area under the curve (AUC) was calculated separately for 8-epi-PGF2α, MDA, and SOD1. Internal validation and estimation of 95% confidence intervals were performed using 1000 bootstrap resamples. The 24-month horizon was selected because it captured most observed survival events while maintaining adequate follow-up in the study cohort. Time-dependent cutoff values were identified using the Youden index, with corresponding sensitivity and specificity. These internally derived thresholds were subsequently used for exploratory Kaplan–Meier stratification and should not be considered externally validated clinical decision limits. At 24 months, 16 of the 19 observed OS events and 30 of the 39 observed PFS events had occurred.

Survival analyses were performed using the Kaplan–Meier method. Patients were stratified according to ROC-derived cutoff values for 8-epi-PGF2α, MDA, and SOD1, and differences between survival curves were assessed using the log-rank (Mantel–Cox) test. These internally derived cutoff values were used to stratify patients for the subsequent Kaplan–Meier analyses. OS was defined as the interval from the date of diagnosis to death from any cause or last follow-up. PFS was defined as the interval from the date of diagnosis to the first documented disease recurrence, disease progression, or death before documented progression, or to the last follow-up for patients without an event. Because the study cohort included patients with metastatic disease at baseline, PFS was used instead of disease-free survival (DFS).

Cox proportional hazards regression analyses were performed to evaluate the association between oxidative stress biomarkers and survival outcomes. Hazard ratios (HRs), 95% confidence intervals (CIs), and *p*-values were reported. Covariates for multivariable modeling were selected a priori based on their established clinical and prognostic relevance in CRC and included age, sex, TNM stage, and metastatic status, as well as 24-month time-dependent ROC-derived MDA status, the oxidative stress biomarker of primary prognostic interest. The number of observed events was taken into account when determining model complexity. For OS, 19 deaths were recorded; because of this limited number of events and the fact that all deaths occurred in patients with metastatic disease, multivariable Cox regression for OS was considered unstable and was therefore not performed. For PFS, 39 events were observed; accordingly, parsimonious multivariable models were constructed to limit the number of covariates relative to the available events and reduce the risk of overfitting. Because TNM stage and metastatic status convey overlapping prognostic information, they were entered into separate multivariable models to reduce collinearity. To assess whether the association between MDA and PFS depended on the dichotomization using an internally derived cutoff, an additional sensitivity analysis was performed with MDA modeled as a continuous variable. For interpretability, MDA was standardized, and the HR was expressed per 1-standard deviation (SD) increase in serum concentration. The same covariate structure as in the cutoff-based PFS models was retained: Model 1 adjusted for age, sex, and TNM stage, whereas Model 2 adjusted for age, sex, and metastatic status. The proportional hazards assumption was assessed using Schoenfeld residuals for both the cutoff-based and continuous-MDA Cox models, with no statistically significant violations detected (all *p* > 0.05).

The primary analyses of the study comprised comparisons of oxidative stress biomarkers between CRC patients and healthy controls, as well as the evaluation of their prognostic relationships with survival outcomes (OS and PFS). Subgroup analyses according to TNM stage, T stage, N stage, M stage, histological grade, and tumor sidedness, as well as correlation analyses, were considered secondary and exploratory. For post hoc multiple comparisons following significant overall group differences, Bonferroni correction was applied. Given the large number of biomarkers, inflammatory indices, subgroup comparisons, and correlation analyses, no single multiplicity adjustment was applied across the entire analytical framework. Accordingly, the broader exploratory analyses remain susceptible to false-positive findings due to multiple testing, and nominally significant *p*-values from these analyses should be interpreted with caution and treated as hypothesis-generating. All statistical tests were two-sided, and a *p*-value < 0.05 was considered statistically significant.

## 3. Results

### 3.1. Baseline Clinicopathological Characteristics

The study included 140 patients diagnosed with CRC and 40 healthy control subjects. The mean age of CRC patients was 65.66 ± 7.60 years, compared with 61.75 ± 13.92 years in the control group; no statistically significant difference was observed between the groups (*p* = 0.095). The CRC group included 56 females and 84 males, whereas the control group included 18 females and 22 males; the sex distribution did not differ significantly between groups (*p* = 0.571). These findings are consistent with the group-level frequency-matching procedure used for age and sex (Table 1).

Regarding tumor extension, most CRC patients presented with advanced local disease, with T3 tumors representing the predominant subgroup (*n* = 89), followed by T4 tumors (*n* = 37). Early-stage tumors were less frequent, with only 6 patients classified as T1 and 8 patients as T2. These findings suggest that the majority of patients were diagnosed in relatively advanced stages of local tumor invasion.

Lymph node involvement was common within the study cohort. While 39 patients presented without nodal metastasis (N0), most patients exhibited regional lymph node dissemination, including 66 with N1 and 35 with N2 disease. Furthermore, distant metastases were identified in 43 patients, whereas 97 patients presented without metastatic disease (M0).

TNM stage distribution demonstrated a predominance of advanced-stage colorectal cancer. Stage III tumors represented the largest subgroup (*n* = 60), followed by stage IV disease (*n* = 43). In contrast, only 14 patients were diagnosed with stage I disease, and 23 patients with stage II tumors. These findings indicate that a substantial proportion of patients were diagnosed with locoregionally advanced or metastatic disease.

Histopathological grading revealed a relatively balanced distribution among tumor differentiation categories, although poorly differentiated tumors (G3, *n* = 55) represented the largest subgroup, followed by well-differentiated tumors (G1, *n* = 44) and moderately differentiated tumors (G2, *n* = 41). Analysis of tumor sidedness demonstrated that left-sided colorectal tumors predominated within the cohort (*n* = 98), whereas right-sided tumors were identified in 42 patients.

### 3.2. Comparison of Circulating Oxidative Stress Biomarkers (8-epi-PGF2α, MDA, and SOD1) in CRC Versus Controls

Table 2 summarizes the circulating oxidative stress biomarkers, hematological parameters, and inflammatory indices in CRC patients and healthy controls. Significant differences were observed in several oxidative stress and hematological variables, indicating both oxidative imbalance and systemic hematological alterations in CRC patients.

Regarding oxidative stress biomarkers, CRC patients exhibited significantly higher serum concentrations of 8-epi-PGF2α compared with controls (381.91 [228.54–584.68] vs. 270.64 [234.83–374.77] pg/mL, *p* = 0.016). More pronounced differences were observed for MDA and SOD1. Median MDA levels were more than threefold higher in CRC patients than in controls (331.05 [242.23–447.26] vs. 92.43 [70.34–115.90] ng/mL, *p* < 0.001), while SOD1 concentrations were also markedly elevated (2993.47 [1741.87–4385.84] vs. 1037.87 [590.14–1761.20] pg/mL, *p* < 0.001). These findings indicate substantial activation of oxidative stress pathways accompanied by increased antioxidant defense activity in CRC. The magnitude of the between-group difference was small-to-moderate for 8-epi-PGF2α (rank-biserial correlation, rrb = 0.249; 95% CI, 0.063–0.443), whereas substantially larger effects were observed for MDA (rrb = 1.000; 95% CI, 1.000–1.000) and SOD1 (rrb = 0.675; 95% CI, 0.553–0.790).

Among hematological parameters, CRC patients had significantly lower Hb levels than controls (10.56 [8.10–12.44] vs. 13.30 [12.60–14.20] g/dL, *p* < 0.001), consistent with the frequent occurrence of anemia in colorectal malignancy. WBC counts were significantly increased in CRC patients (8.35 [6.84–9.98] vs. 6.61 [5.26–8.40] ×10^3^/μL, *p* < 0.001), as were NEU counts (5.13 [4.07–6.67] vs. 3.90 [2.81–5.93] ×10^3^/μL, *p* = 0.004). Interestingly, LYM counts were also modestly but significantly higher in CRC patients (2.11 [1.57–2.78] vs. 1.48 [1.16–2.12] ×10^3^/μL, *p* = 0.010). In contrast, MON and PLT counts did not differ significantly between groups (both *p* > 0.05).

No significant differences were observed in erythrocyte indices, including MCV, MCH, MCHC, and RDW (all *p* > 0.05), suggesting that the observed hematological alterations were predominantly related to inflammatory and immune cell compartments rather than to RBC morphology.

Among inflammatory biomarkers, MLR was significantly lower in CRC patients compared with controls (0.266 [0.196–0.355] vs. 0.402 [0.281–0.573], *p* < 0.001), while MCVL was also significantly reduced in the CRC group (40.82 [31.06–59.39] vs. 59.93 [46.74–60.33], *p* = 0.037). In contrast, NLR, PLR, AISI, SII, SIRI, and IIC did not demonstrate statistically significant differences between CRC patients and controls (all *p* > 0.05). Overall, the results indicate that CRC is characterized by a marked increase in oxidative stress biomarkers, particularly MDA and SOD1, accompanied by anemia, leukocytosis, neutrophilia, and alterations in selected inflammatory indices. Among the evaluated inflammatory markers, MLR and MCVL emerged as the indices most strongly differentiating CRC patients from healthy controls.

### 3.3. Oxidative Stress Biomarkers According to TNM Stage

To investigate differences in oxidative stress biomarkers, hematological parameters, and inflammatory indices according to disease stage, these parameters were compared across TNM stages in CRC patients. The results are summarized in Table 3.

Significant overall differences were observed for all three oxidative stress biomarkers across TNM categories. Serum concentrations of 8-epi-PGF2α (*p* < 0.001), MDA (*p* < 0.001), and SOD1 (*p* < 0.001) differed significantly across disease stages according to the Kruskal–Wallis analysis. However, their distributions were not strictly linear across TNM stages. The highest concentrations were generally observed in stage II and stage IV disease, whereas stage III tumors frequently demonstrated lower values. Therefore, these overall differences should not be interpreted as evidence of a consistent progressive increase in oxidative stress biomarkers with advancing TNM stage.

Among the investigated biomarkers, MDA showed marked differences across TNM stage. Patients with stage II disease exhibited the highest MDA concentrations (600.00 [343.36–619.88] ng/mL), followed by stage IV tumors (538.95 [489.73–612.78] ng/mL), whereas substantially lower levels were observed in stage III disease (244.53 [231.25–276.79] ng/mL; overall *p* < 0.001). Similarly, SOD1 concentrations differed significantly across TNM stages, with higher median values in stage II and stage IV tumors (3398.69 [1819.15–3898.69] pg/mL and 3398.69 [3215.68–3712.41] pg/mL, respectively) than in stage III disease (1973.99 [1607.96–2467.58] pg/mL; overall *p* < 0.001). Significant overall differences were also observed for 8-epi-PGF2α (*p* < 0.001), with the highest median values detected in stage IV tumors (413.91 [391.11–456.95] pg/mL). Specific statistically significant pairwise differences are reported separately in Section 3.4 and Table 4.

Among conventional hematological parameters, LYM counts differed significantly across TNM stages (overall *p* < 0.001). The highest median LYM values were observed in stage III disease (2.60 [1.68–3.24] ×10^3^/μL), whereas stage IV patients presented substantially lower counts (1.90 [1.31–2.38] ×10^3^/μL). RDW also demonstrated significant overall variation across TNM stages (*p* = 0.010), with higher median values in stage IV tumors (13.90 [13.04–17.46] %) than in stage III disease (13.04 [12.30–14.12] %). In contrast, Hb, WBC, NEU, MON, PLT count, MCV, MCH, and MCHC did not differ significantly across TNM stages (all *p* > 0.05).

Several inflammatory biomarkers differed significantly across TNM stages. NLR showed significant overall differences (*p* = 0.002), with the highest median value observed in stage IV disease (2.97 [2.21–4.21]). However, NLR did not show a progressive increase across stages. PLR also differed significantly across TNM stages (*p* < 0.001), with relatively higher median values observed in stage I (152.44 [131.60–290.03]) and stage IV disease (135.19 [115.33–214.11]). Similarly, MLR varied significantly across TNM stages (*p* = 0.006).

Composite inflammatory indices also showed significant overall differences across TNM stages. AISI (*p* = 0.001), SII (*p* = 0.002), SIRI (*p* = 0.003), and IIC (*p* = 0.001) differed significantly across TNM groups. Stage IV tumors exhibited relatively high median values for SII (854.11 [536.25–1154.93]), SIRI (1.91 [1.15–2.75]), and IIC (3.34 [2.53–6.18]); however, the distributions of these indices across the four TNM stages were not uniformly progressive.

Notably, MCVL also differed significantly across TNM stages (*p* = 0.006). Lower median MCVL values were observed in stage II and stage III disease (34.93 [31.46–46.87] and 37.71 [26.62–47.01], respectively), whereas higher values were observed in stage I and stage IV tumors, further illustrating the non-linear patterns observed across TNM categories.

Overall, the Kruskal–Wallis analyses identified significant differences across TNM stages for all three oxidative stress biomarkers and for multiple inflammatory indices, including NLR, PLR, MLR, AISI, SII, SIRI, MCVL, and IIC. Importantly, these significant overall differences did not represent a uniform monotonic increase with advancing TNM stage. The specific stage-to-stage differences responsible for the significant omnibus results were therefore further examined using pairwise post hoc comparisons with Bonferroni correction, as presented in Section 3.4 and Table 4.

### 3.4. Pairwise Mann–Whitney U Post-Hoc Analysis with Bonferroni Correction

Because significant overall differences were identified by Kruskal–Wallis analyses across TNM stages for several oxidative stress biomarkers and inflammatory indices, pairwise post hoc analyses with Bonferroni correction were performed to identify the specific stage comparisons underlying these overall differences. The significant pairwise comparisons are presented in Table 4.

Post hoc pairwise comparisons showed that many of the significant differences involved stage III and stage IV disease. For oxidative stress biomarkers, significantly higher concentrations of 8-epi-PGF2α and SOD1 were observed in stage IV compared with stage III tumors (both adjusted *p* < 0.001). MDA showed significant pairwise differences between stages I and III, I and IV, II and III, and III and IV (all adjusted *p* < 0.001).

Among hematological parameters, LYM counts were significantly lower in stage IV than in stage II (adjusted *p* = 0.015) and stage III (adjusted *p* = 0.001), whereas RDW was significantly higher in stage IV than in stage III (adjusted *p* = 0.005).

Regarding inflammatory biomarkers, the most consistent differences were observed between stage III and stage IV disease. Stage IV tumors exhibited significantly higher NLR, PLR, MLR, AISI, SII, SIRI, MCVL, and IIC values compared with stage III tumors. Additional significant differences were identified between stages II and III for AISI and SII, and between stages I and III for PLR.

Collectively, the post hoc analyses identified stage III versus stage IV as the most frequent significant pairwise comparison for several oxidative stress biomarkers and inflammatory indices. These findings specify the stage comparisons underlying the significant Kruskal–Wallis omnibus results without implying a uniform progressive increase across all TNM stages.

### 3.5. Oxidative Stress Biomarkers According to T Stage

To evaluate the relationship between local tumor invasion and systemic oxidative–inflammatory status, oxidative stress biomarkers, hematological parameters, and inflammatory indices were compared according to T stage. Patients were classified into T1 + T2, T3, and T4 groups, and the results are summarized in Table 5.

The comparison by T stage revealed significant differences in several hematological and inflammatory biomarkers, whereas oxidative stress markers did not show statistically significant variation across categories of local tumor invasion.

Serum concentrations of 8-epi-PGF2α, MDA, and SOD1 were comparable among T-stage groups (all *p* > 0.05). Although T4 tumors exhibited higher median MDA levels (553.71 [239.84–614.41] ng/mL) compared with T1 + T2 and T3 lesions, the overall difference did not reach statistical significance (*p* = 0.076).

Among hematological parameters, Hb levels differed significantly according to tumor invasion depth (*p* = 0.045), with the highest values observed in T4 tumors (11.59 [8.80–13.40] g/dL). LYM counts also showed significant variation (*p* = 0.017), with T4 tumors exhibiting higher values (2.60 [2.02–3.04] ×10^3^/μL) than T1 + T2 disease (1.79 [1.50–2.34] ×10^3^/μL).

Among inflammatory biomarkers, significant differences were observed for PLR (*p* = 0.025), MLR (*p* = 0.006), and MCVL (*p* = 0.030). PLR progressively decreased with increasing tumor invasion, from 141.33 [131.20–193.55] in T1 + T2 disease to 107.53 [90.77–127.31] in T4 tumors. Similarly, MLR decreased from 0.29 [0.18–0.42] in early-stage tumors to 0.21 [0.15–0.29] in T4 lesions. MCVL also showed lower values in T4 disease (35.92 [27.27–44.97]) than in T1 + T2 tumors (49.37 [38.29–60.44]).

Overall, local tumor invasion was primarily associated with alterations in selected inflammatory biomarkers rather than substantial changes in circulating oxidative stress markers.

### 3.6. Oxidative Stress Biomarkers According to N Stage

To investigate the impact of lymph node involvement on oxidative stress and systemic inflammatory activation, biomarkers were subsequently analyzed according to nodal status (N0–N2). The results are presented in Table 6.

Nodal involvement was associated with significant alterations in selected oxidative stress and inflammatory biomarkers.

Among oxidative stress markers, MDA was the only biomarker demonstrating significant differences across N stages (*p* = 0.002). Patients without nodal involvement (N0) exhibited high MDA concentrations (486.45 [321.29–606.02] ng/mL), whereas N1 disease showed lower levels (283.28 [240.14–463.27] ng/mL). Elevated values were again observed in N2 disease (479.06 [237.11–612.78] ng/mL), suggesting a non-linear relationship between lipid peroxidation and nodal progression. In contrast, 8-epi-PGF2α and SOD1 concentrations did not differ significantly according to nodal status (both *p* > 0.05).

Among hematological parameters, PLT counts demonstrated significant variation across N stages (*p* = 0.047), with the highest values observed in N0 disease (297.30 [236.50–373.70] ×10^3^/μL). No significant differences were observed for Hb, WBC, NEU, LYM, MON count, or erythrocyte indices.

Among inflammatory biomarkers, AISI was the only index showing a nominally significant difference across nodal categories (*p* = 0.049). The highest AISI values were observed in N0 patients (541.93 [257.73–898.31]), while lower values were detected in N1 and N2 disease. Other inflammatory indices, including NLR, PLR, MLR, SII, SIRI, MCVL, and IIC, did not differ significantly between nodal categories.

Overall, differences in oxidative stress and inflammatory parameters across nodal categories were less pronounced than those observed across TNM or metastatic-status groups. Among the oxidative stress biomarkers, MDA showed the most evident differences across N-stage categories.

### 3.7. Oxidative Stress Biomarkers According to M Stage

Because distant metastasis is one of the strongest prognostic determinants in CRC, oxidative stress biomarkers and inflammatory indices were further compared between non-metastatic (M0) and metastatic (M1) patients. The results are presented in Table 7.

Marked differences in oxidative stress biomarkers and systemic inflammatory indices were observed between patients with non-metastatic (M0) and metastatic (M1) disease, representing one of the most pronounced clinicopathological group differences identified in the present study.

All three oxidative stress biomarkers were significantly elevated in patients with metastatic disease. Median 8-epi-PGF2α concentrations were markedly higher in M1 disease compared with M0 tumors (413.91 [391.11–456.95] vs. 260.37 [178.70–443.27] pg/mL, *p* < 0.001). Similarly, MDA levels were substantially increased in metastatic disease (538.95 [489.73–612.78] vs. 263.67 [232.69–344.92] ng/mL, *p* < 0.001). SOD1 concentrations also demonstrated a significant increase in M1 patients (3398.69 [3215.68–3712.41] vs. 1946.81 [1648.69–3503.27] pg/mL, *p* < 0.001).

Among hematological parameters, metastatic patients exhibited significantly lower lymphocyte counts (1.90 [1.31–2.38] vs. 2.40 [1.68–3.08] ×10^3^/μL, *p* < 0.001) and significantly higher RDW values (13.90 [13.04–17.46] vs. 13.10 [12.33–14.30] %, *p* = 0.002). Other conventional hematological parameters did not differ significantly between groups.

Marked differences were also observed for inflammatory indices. Metastatic patients demonstrated significantly higher NLR (*p* < 0.001), PLR (*p* = 0.002), MLR (*p* = 0.006), AISI (*p* = 0.011), SII (*p* = 0.009), SIRI (*p* = 0.006), and IIC (*p* = 0.001) values compared with non-metastatic patients. In addition, MCVL was significantly increased in M1 disease (47.42 [35.50–68.75] vs. 38.68 [28.17–51.69], *p* = 0.004).

Collectively, these findings indicate that metastatic CRC is characterized by pronounced oxidative stress activation accompanied by substantial systemic inflammatory dysregulation. The simultaneous elevation of 8-epi-PGF2α, MDA, SOD1, and multiple inflammatory indices suggests that oxidative stress and inflammation may act synergistically during metastatic progression.

### 3.8. Oxidative Stress Biomarkers According to Tumor Grade

To further investigate the relationship between tumor differentiation and oxidative–inflammatory status, oxidative stress biomarkers, hematological parameters, and inflammatory indices were compared across histopathological tumor grades (G1–G3). The results are presented in Table 8.

Tumor grade analysis revealed significant differences in oxidative stress biomarkers and inflammatory indices across histological grades.

Among the oxidative stress biomarkers, MDA demonstrated significant variation across tumor grades (*p* = 0.015). The highest MDA concentrations were observed in G2 tumors (463.48 [267.92–560.27] ng/mL), followed by G1 lesions (446.25 [263.57–576.20] ng/mL), whereas G3 tumors exhibited substantially lower values (256.64 [231.60–479.47] ng/mL). Similarly, SOD1 levels differed significantly by tumor differentiation (*p* = 0.005), with higher concentrations observed in G1 and G2 tumors than in G3 lesions. In contrast, 8-epi-PGF2α concentrations were comparable among the three histological grades (*p* = 0.894).

Among conventional hematological parameters, significant differences were identified for NEU (*p* = 0.014), LYM (*p* = 0.033), MON counts (*p* = 0.010), and RDW (*p* = 0.007). G1 and G2 tumors exhibited higher NEU and MON counts than G3 lesions, while LYM counts were highest in G3 tumors (2.39 [1.63–3.20] ×10^3^/μL). RDW values were highest in moderately differentiated tumors (G2: 14.40 [12.99–17.42] %).

Several inflammatory biomarkers demonstrated strong associations with tumor grade. NLR (*p* = 0.001), PLR (*p* = 0.010), MLR (*p* < 0.001), AISI (*p* < 0.001), SII (*p* < 0.001), SIRI (*p* < 0.001), and IIC (*p* < 0.001) all differed significantly among histological grades. Notably, G1 and G2 tumors consistently exhibited higher inflammatory index values compared with G3 lesions. For example, median SII values were 716.08 [469.53–1212.67] in G1 tumors and 854.11 [582.65–1172.50] in G2 tumors, compared with only 504.94 [374.38–638.28] in G3 disease. Similarly, SIRI values were substantially higher in G1 and G2 tumors than in poorly differentiated tumors.

The novel inflammatory biomarker IIC also showed a strong association with tumor grade (*p* < 0.001), with the highest values in G2 tumors (3.30 [2.73–5.84]) and the lowest in G3 lesions (2.27 [1.78–3.22]). In contrast, MCVL did not reach statistical significance (*p* = 0.058), although a trend toward higher values in G2 tumors was observed.

Overall, tumor grade was significantly associated with multiple inflammatory biomarkers and with MDA and SOD1 concentrations. Interestingly, the highest oxidative stress and inflammatory burden were generally observed in G1 and G2 tumors rather than in poorly differentiated G3 lesions, suggesting that the relationship between histological differentiation and systemic oxidative–inflammatory activation may be more complex than a simple linear increase with tumor aggressiveness. This finding further supports the biological heterogeneity of CRC and highlights the importance of integrating multiple biomarkers when assessing tumor behavior and prognosis.

### 3.9. Pairwise Mann–Whitney U Post-Hoc Analysis According to Tumor Grade

Because significant overall differences were identified for several oxidative stress biomarkers and inflammatory indices by tumor grade, pairwise Mann–Whitney U post hoc analyses with Bonferroni correction were subsequently performed to identify the specific histological grade comparisons responsible for these associations.

Post hoc pairwise comparisons revealed that most significant differences were driven by comparisons involving poorly differentiated (G3) tumors. In contrast, relatively few significant differences were observed between G1 and G2 lesions, indicating that the principal biological distinction occurred between poorly differentiated tumors and the remaining histological grades (Table 9).

Among oxidative stress biomarkers, MDA differed significantly between G2 and G3 tumors (adjusted *p* = 0.009), while SOD1 differed significantly between G1 and G3 (adjusted *p* = 0.011) and between G2 and G3 (adjusted *p* = 0.004). These findings indicate that poorly differentiated tumors exhibit a distinct oxidative stress profile compared with well- and moderately differentiated lesions.

Among inflammatory biomarkers, the biggest differences were observed for NLR, MLR, AISI, SII, SIRI, and IIC. For each of these indices, significant differences were identified between G2 and G3 tumors, while several also differed significantly between G1 and G3 disease. Notably, SII, SIRI, AISI, and IIC showed highly significant differences between G2 and G3 tumors (all adjusted *p* < 0.001), suggesting that systemic inflammatory activation varies substantially with histological differentiation.

PLR and NEU counts also differed significantly between G2 and G3 tumors, although the magnitude of these differences was less pronounced than for the composite inflammatory indices.

Interestingly, LYM and MON counts, RDW, and MCVL lost statistical significance after Bonferroni correction, despite demonstrating significant overall results in the Kruskal–Wallis test. This finding suggests that the observed global associations were distributed across multiple groups and were insufficiently strong to remain significant after adjustment for multiple testing.

Overall, the post hoc analysis demonstrated that poorly differentiated tumors are the subgroup most distinct from the other histological grades, particularly in inflammatory activation and antioxidant response. The predominance of G2–G3 differences suggests that tumor dedifferentiation is accompanied by substantial alterations in systemic inflammatory pathways and oxidative stress regulation, further supporting the biological relevance of these biomarkers in CRC progression.

### 3.10. Oxidative Stress Biomarkers According to Tumor Sidedness

To evaluate whether tumor sidedness influences oxidative stress and systemic inflammatory activation, oxidative stress biomarkers, hematological parameters, and inflammatory indices were compared between right-sided and left-sided colorectal tumors. The results are presented in Table 10.

Tumor sidedness analysis revealed limited differences between right-sided and left-sided colorectal tumors. Among oxidative stress biomarkers, SOD1 was significantly higher in right-sided tumors compared with left-sided lesions (3398.70 [1957.45–3777.78] vs. 2095.74 [1715.43–3424.84] pg/mL, *p* = 0.012), suggesting higher circulating SOD1 levels in patients with proximal colorectal tumors. MDA tended to be higher in right-sided tumors, but this difference did not reach statistical significance (476.84 [256.75–607.71] vs. 315.62 [238.77–508.89] ng/mL, *p* = 0.094). 8-epi-PGF2α levels were comparable between groups (*p* = 0.754).

Among hematological parameters, RDW was significantly higher in right-sided tumors than in left-sided tumors (14.45 [13.07–17.25] vs. 13.10 [12.34–14.10], *p* < 0.001). No significant differences were observed for Hb, WBC, NEU, LYM, MON, PLT, MCV, MCH, or MCHC.

Inflammatory indices, including NLR, PLR, MLR, AISI, SII, SIRI, MCVL, and IIC, did not differ significantly by tumor sidedness. Overall, these findings suggest that tumor location is primarily associated with circulating SOD1 concentrations and RDW alterations, while most systemic inflammatory indices remain comparable between right- and left-sided CRC.

### 3.11. Prognostic Significance of Oxidative Stress Biomarkers

A total of 140 patients were included in the survival analyses. The median follow-up duration for OS was 19.2 months (range, 1.3–34.6 months), during which 19 deaths were recorded, and 121 patients were censored. For PFS, the median follow-up was 19.6 months (range, 1.7–35.1 months), with 39 PFS events (disease recurrence, disease progression, or death before documented progression) and 101 censored patients.

#### 3.11.1. Time-Dependent ROC Analysis for OS and PFS

To account for censoring in the time-to-event data, the discriminatory performance of the three oxidative stress biomarkers was evaluated using time-dependent ROC analysis at a predefined 24-month prediction horizon (Table 11). The 24-month time point was selected because 16 of the 19 observed deaths and 30 of the 39 PFS events had occurred by this time, while sufficient follow-up remained available within the cohort. Internal validation and estimation of 95% confidence intervals were performed using 1000 bootstrap resamples.

For OS at 24 months, MDA demonstrated the highest discriminative performance, with a time-dependent AUC of 0.920 (95% bootstrap CI: 0.834–0.978). The corresponding internally derived cutoff was 538.95 ng/mL, providing a sensitivity of 100.0% and a specificity of 75.0%. SOD1 showed substantially lower discrimination, with an AUC of 0.665 (95% CI: 0.501–0.805), whereas 8-epi-PGF2α showed limited discriminatory ability, with an AUC of 0.564 (95% CI: 0.400–0.722).

For PFS at 24 months, MDA again showed the highest AUC among the three biomarkers, although its discriminatory performance was considerably more modest than that observed for OS (AUC = 0.636; bootstrap 95% CI: 0.487–0.773). The corresponding cutoff was 552.89 ng/mL, with a sensitivity of 56.9% and specificity of 76.2%. 8-epi-PGF2α demonstrated limited discrimination (AUC = 0.548; 95% CI: 0.395–0.679), whereas SOD1 showed no meaningful discriminatory performance for 24-month PFS (AUC = 0.423; 95% CI: 0.289–0.549).

Overall, the time-dependent analysis identified MDA as the biomarker with the highest discriminatory performance for both endpoints in this cohort, particularly for 24-month OS. However, the substantially lower AUC for PFS and the bootstrap confidence interval encompassing 0.50 indicate that its ability to discriminate 24-month progression risk was limited. Furthermore, because these estimates and cutoff values were internally derived from the same cohort, they should be considered exploratory and require external validation.

#### 3.11.2. Kaplan–Meier Analysis for Overall Survival

Kaplan–Meier analyses were subsequently performed using the internally derived 24-month time-dependent ROC cutoff values to explore differences in OS between patients with lower and higher circulating biomarker concentrations. These analyses should be considered exploratory because the cutoff values were derived and evaluated within the same study cohort.

**Figure 2:**
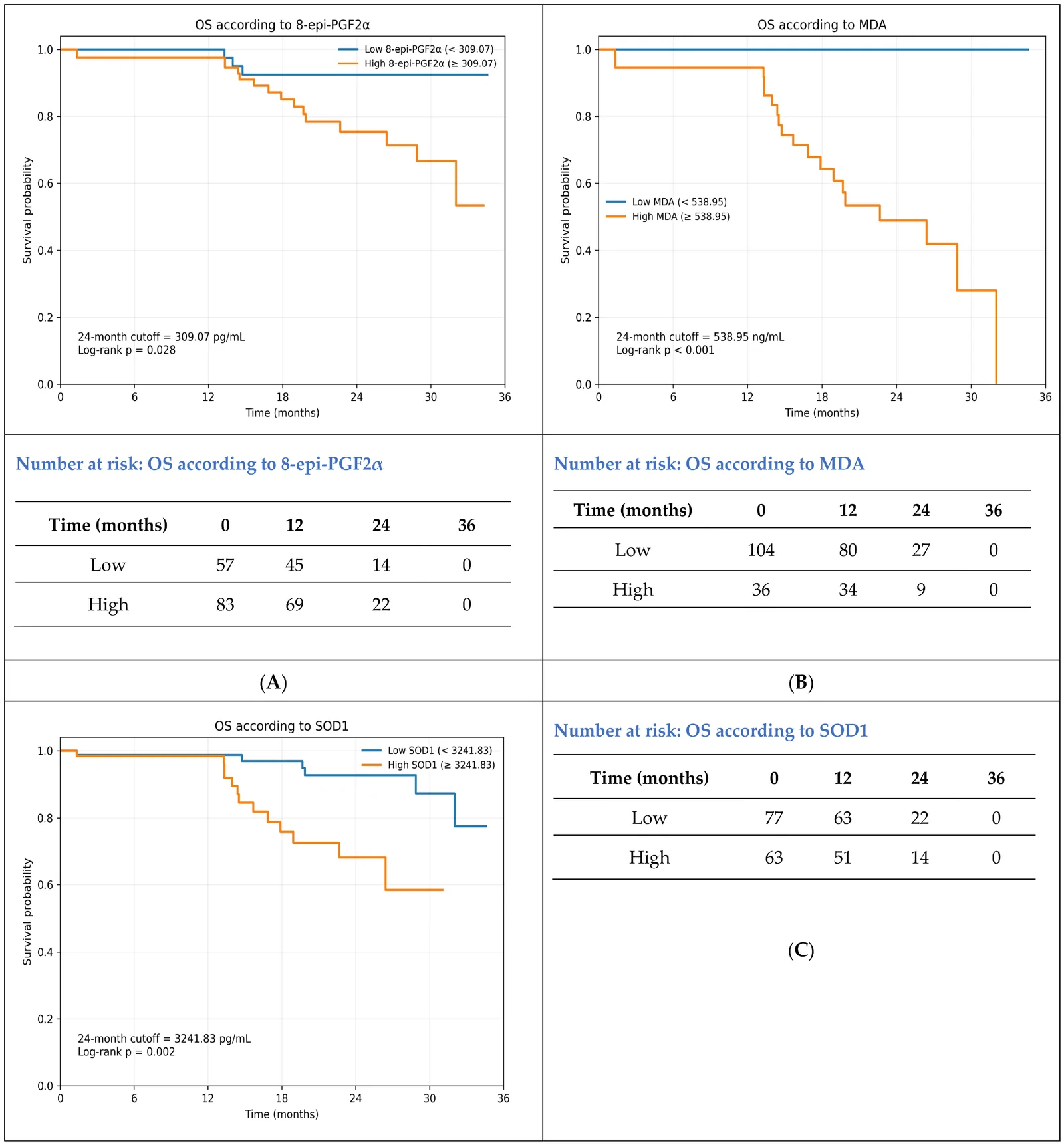
Kaplan–Meier overall survival (OS) analysis stratified according to the internally derived 24-month time-dependent ROC cutoff values of serum 8-epi-PGF2α (**A**), MDA (**B**), and SOD1 levels (**C**). A. Patients were categorized into lower and higher biomarker groups according to the corresponding time-dependent cutoff values, and survival distributions were compared using the log-rank test. Numbers at risk at 0, 12, 24, and 36 months are provided for each biomarker-stratified analysis. (**A**) 8-epi-PGF2α cutoff: 309.07 pg/mL, log-rank *p* = 0.008. (**B**) MDA cutoff: 538.95 ng/mL, log-rank *p* < 0.001. (**C**) SOD1 cutoff: 3241.83 pg/mL, log-rank *p* = 0.002. The cutoff values were derived internally using time-dependent ROC analysis with a predefined 24-month prediction horizon and should be considered exploratory, pending external validation.

For 8-epi-PGF2α, patients with concentrations above the time-dependent cutoff of 309.07 pg/mL showed significantly shorter OS than those with concentrations below this threshold (log-rank *p* = 0.008). Similarly, higher SOD1 concentrations, defined using the cutoff of 3241.83 pg/mL, were associated with significantly shorter OS (log-rank *p* = 0.002) (Table 11, Figure 2).

The strongest separation of the Kaplan–Meier survival curves was observed for MDA. Patients with MDA concentrations ≥538.95 ng/mL had significantly shorter OS than those below this threshold (log-rank *p* < 0.001), consistent with MDA’s comparatively high discriminative performance observed in the 24-month time-dependent ROC analysis. Nevertheless, this finding should be interpreted cautiously because only 19 deaths occurred during follow-up, all among patients with metastatic disease, and the MDA threshold was derived and evaluated within the same cohort (Table 11).

Overall, the Kaplan–Meier analyses showed significant OS differences according to the internally derived thresholds for all three oxidative stress biomarkers, with the most pronounced separation observed for MDA. These findings are exploratory and require validation in independent cohorts.

#### 3.11.3. Kaplan–Meier Progression-Free Survival Analysis

**Figure 3:**
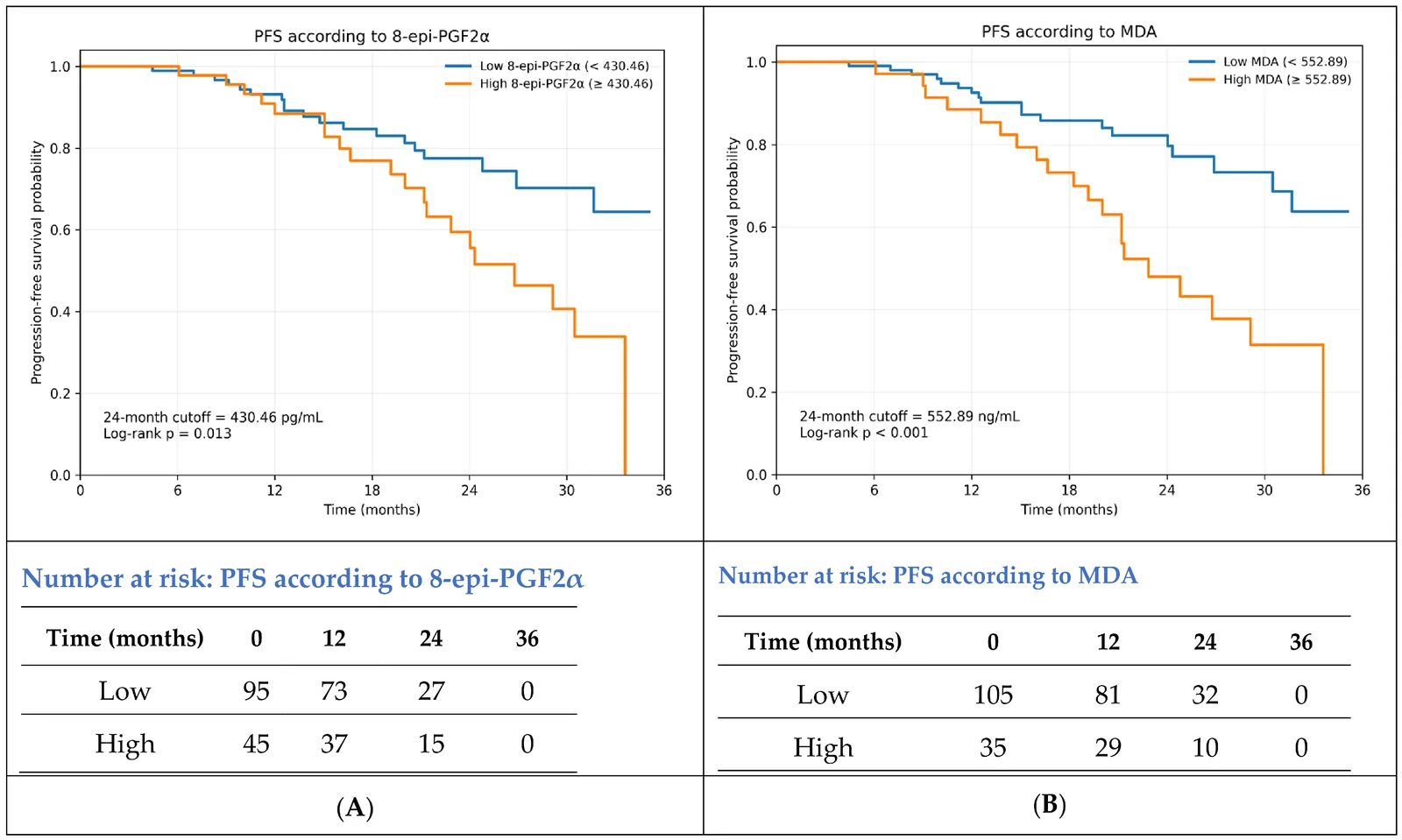

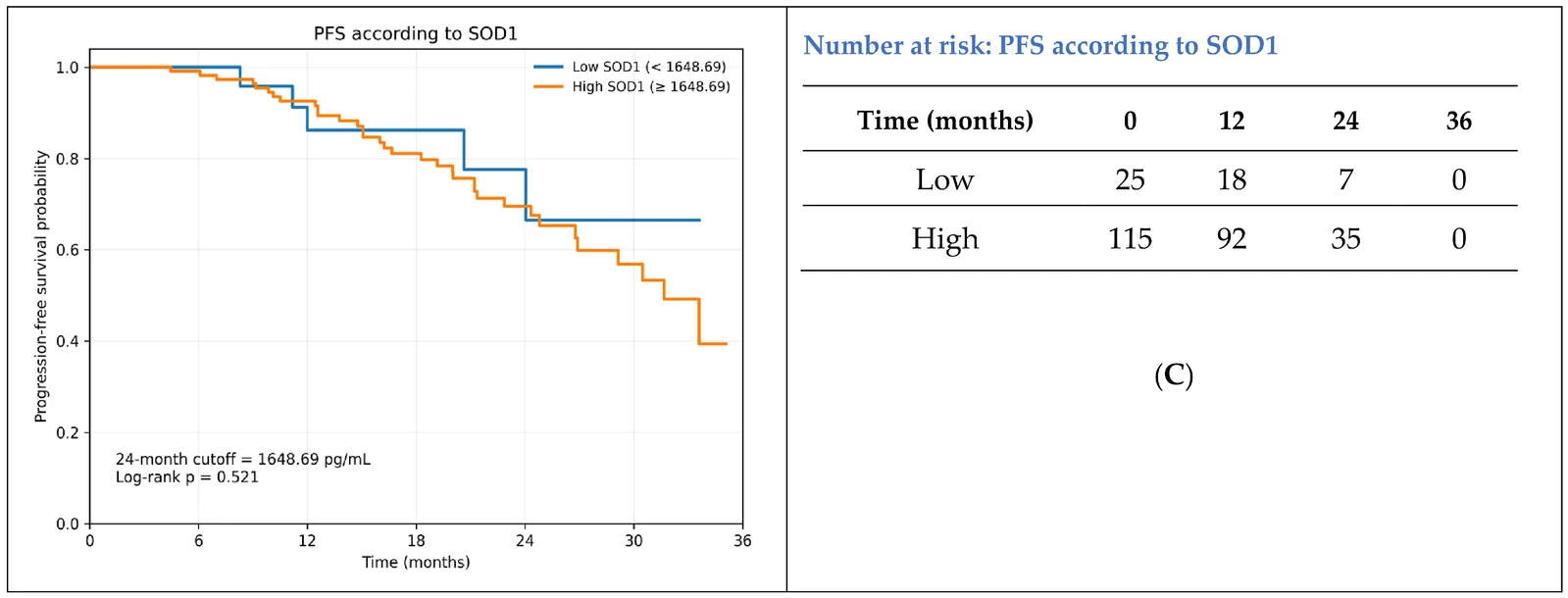
Kaplan–Meier progression-free survival (PFS) analyses stratified according to the internally derived 24-month time-dependent ROC cutoff values for serum 8-epi-PGF2α (**A**), MDA (**B**), and SOD1 (**C**). Patients were categorized into lower- and higher-biomarker groups according to the corresponding time-dependent cutoff values, and survival distributions were compared using the log-rank test. Numbers at risk at 0, 12, 24, and 36 months are provided for each biomarker-stratified analysis. (**A**) 8-epi-PGF2α cutoff: 430.46 pg/mL, log-rank *p* = 0.013. (**B**) MDA cutoff: 552.89 ng/mL, log-rank *p* < 0.001. (**C**) SOD1 cutoff: 1648.69 pg/mL, log-rank *p* = 0.521. The cutoff values were derived internally using time-dependent ROC analysis with a predefined 24-month prediction horizon and should be considered exploratory, pending external validation.

Kaplan–Meier analyses were subsequently performed using the internally derived 24-month time-dependent ROC cutoff values to assess differences in PFS by circulating oxidative stress biomarker concentrations. Because these thresholds were derived and evaluated within the same cohort, the Kaplan–Meier analyses should be considered exploratory (Table 11, Figure 3).

Patients with serum 8-epi-PGF2α concentrations ≥430.46 pg/mL had significantly shorter PFS than those with concentrations below this threshold (log-rank *p* = 0.013). However, the corresponding 24-month time-dependent AUC was only 0.548 (95% bootstrap CI: 0.395–0.679), indicating limited standalone discriminative performance despite the significant separation of the Kaplan–Meier curves (Table 11, Figure 3).

MDA showed the most pronounced PFS separation among the investigated biomarkers. Patients with serum MDA concentrations ≥552.89 ng/mL had significantly shorter PFS than those with lower concentrations (log-rank *p* < 0.001). Nevertheless, the time-dependent discriminatory performance of MDA for 24-month PFS was modest (AUC = 0.636; bootstrap 95% CI: 0.487–0.773). Thus, the significant Kaplan–Meier separation should not be interpreted as evidence of strong individual discrimination for 24-month PFS (Table 11).

In contrast, PFS did not differ significantly according to the internally derived SOD1 cutoff of 1648.69 pg/mL (log-rank *p* = 0.521), consistent with the absence of meaningful discriminatory performance in the 24-month time-dependent ROC analysis (AUC = 0.423; bootstrap 95% CI: 0.289–0.549) (Table 11).

Overall, significant PFS differences were observed for 8-epi-PGF2α and MDA, with the strongest Kaplan–Meier separation observed for MDA. However, the time-dependent ROC results indicate that the ability of these biomarkers to discriminate 24-month PFS was limited, and these internally derived findings require external validation.

#### 3.11.4. Cox Proportional Hazards Regression Analysis

Cox proportional hazards regression analyses were performed to further evaluate the prognostic significance of oxidative stress biomarkers. In univariate Cox analysis for OS, elevated 8-epi-PGF2α and SOD1 levels were significantly associated with increased mortality risk. Because all deaths occurred in metastatic patients and the number of OS events was limited, multivariable Cox regression for OS was not performed to avoid model instability and overfitting (Table 12).

For PFS, high MDA levels, defined according to the internally derived 24-month time-dependent ROC cutoff of 552.89 ng/mL, were significantly associated with an increased risk of recurrence, progression, or death. This association remained significant after adjustment for age, sex, and TNM stage (HR = 2.49, 95% CI: 1.29–4.80, *p* = 0.006). Similarly, high MDA remained significantly associated with poorer PFS after adjustment for age, sex, and metastatic status (HR = 2.40; 95% CI, 1.22–4.72; *p* = 0.011). These findings indicate that the association between high MDA and PFS was not fully accounted for by the clinicopathological covariates included in the respective models. However, given the observational design and the absence of adjustment for all potentially relevant prognostic factors, these results should be interpreted as evidence of a statistical prognostic association rather than as evidence that MDA is an independently validated prognostic biomarker.

To evaluate the robustness of this association without relying on dichotomization at an internally derived cutoff, MDA was additionally analyzed as a continuous variable standardized per 1-SD increase (255.19 ng/mL). In univariable Cox regression, each 1-SD increase in MDA was significantly associated with a higher hazard of progression (HR = 1.51, 95% CI: 1.20–1.88, *p* < 0.001). However, the association was attenuated and was no longer statistically significant after adjustment for age, sex, and TNM stage (HR = 1.18, 95% CI: 0.90–1.53, *p* = 0.234) or after adjustment for age, sex, and metastatic status (HR = 1.22, 95% CI: 0.92–1.63, *p* = 0.172). Thus, the results of the continuous-variable sensitivity analysis were not fully concordant with those obtained using the dichotomized MDA threshold, indicating that the prognostic association between MDA and PFS is sensitive to the method used to model MDA and should be interpreted cautiously.

### 3.12. Correlation Analysis Between Oxidative Stress Biomarkers and Hematological Inflammatory Indices

**Figure 4:**
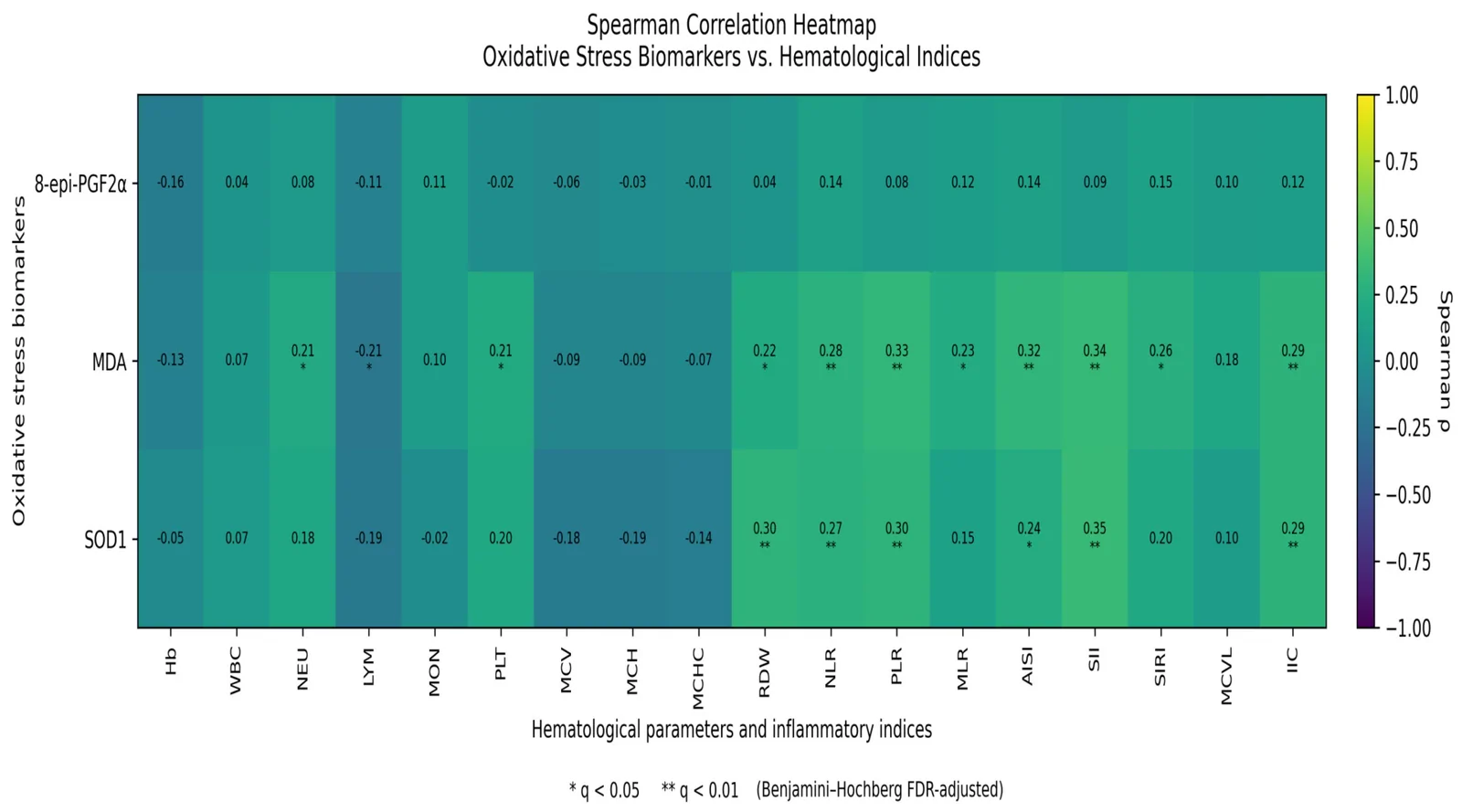
Heatmap of Spearman correlations between oxidative stress biomarkers and hematological inflammatory indices. Cell values represent Spearman correlation coefficients (ρ), and statistical significance is assessed using Benjamini–Hochberg FDR-adjusted *q*-values. After FDR correction, MDA retained the broadest pattern of significant correlations, particularly with composite inflammatory indices including NLR, PLR, AISI, SII, SIRI, and IIC. SOD1 retained significant correlations with RDW, NLR, PLR, AISI, SII, and IIC, whereas 8-epi-PGF2α showed no significant correlations. The largest correlation coefficient was observed between SOD1 and SII (ρ = 0.350). Overall, the significant correlations were predominantly weak to moderate in magnitude and should not be interpreted as evidence of directionality or causality. *q* < 0.05; *q* < 0.01; *q* < 0.001, after Benjamini–Hochberg FDR correction.

To further investigate the relationship between systemic oxidative stress and inflammatory activation in patients with CRC, Spearman correlation analyses were performed between oxidative stress biomarkers (8-epi-PGF2α, MDA, SOD1), conventional hematological parameters, and composite inflammatory biomarkers, including NLR, PLR, MLR, SII, SIRI, AISI, MCVL, and IIC. Given the number of correlation tests performed, the Benjamini–Hochberg procedure was applied to control the false discovery rate (FDR), with *q* < 0.05 considered statistically significant. The correlation matrix, including nominal *p*-values and FDR-adjusted *q*-values, is presented in Table 13 and graphically illustrated in Figure 4.

After FDR correction, distinct correlation patterns remained evident among the three oxidative stress biomarkers. MDA showed the largest number of significant associations with hematological and inflammatory parameters, whereas 8-epi-PGF2α showed no statistically significant correlations after multiple testing correction.

MDA remained significantly positively correlated after FDR correction with NEU count (ρ = 0.210), PLT count (ρ = 0.215), RDW (ρ = 0.220), NLR (ρ = 0.277), PLR (ρ = 0.326), MLR (ρ = 0.230), AISI (ρ = 0.319), SII (ρ = 0.336), SIRI (ρ = 0.256), and IIC (ρ = 0.294), and negatively correlated with LYM count (ρ = −0.209), with all corresponding *q*-values < 0.05. Among the composite inflammatory indices, the largest correlation coefficients were observed for SII, PLR, AISI, and IIC. In contrast, the nominal association between MDA and MCVL (ρ = 0.178, *p* = 0.035) did not remain statistically significant after FDR correction (*q* = 0.079). Overall, the significant MDA correlations were predominantly weak to moderate.

SOD1 showed a more restricted correlation profile after FDR correction. Significant positive correlations remained with RDW (ρ = 0.300), NLR (ρ = 0.270), PLR (ρ = 0.304), AISI (ρ = 0.239), SII (ρ = 0.350), and IIC (ρ = 0.291), with all corresponding *q*-values < 0.05. The largest coefficient was observed between SOD1 and SII (ρ = 0.350), representing a moderate positive correlation. In contrast, the nominal correlations of SOD1 with NEU, LYM, PLT, MCV, MCH, and SIRI did not remain statistically significant after FDR correction.

In contrast, 8-epi-PGF2α demonstrated no statistically significant correlations with the investigated hematological parameters or inflammatory indices after FDR correction.

Overall, after correction for multiple testing, MDA retained the broadest correlation profile with hematological and inflammatory parameters, followed by SOD1, whereas 8-epi-PGF2α showed no significant correlations. The predominantly weak-to-moderate effect sizes indicate statistical covariation rather than evidence of a strong or causal relationship between oxidative stress and systemic inflammation.

## 4. Discussion

The present study comprehensively evaluated oxidative stress biomarkers and hematological inflammatory indices in patients with CRC and explored their associations with clinicopathological characteristics and survival outcomes. The principal findings were as follows: (i) serum levels of 8-epi-PGF2α, MDA, and SOD1 were significantly elevated in CRC patients compared with healthy controls; (ii) oxidative stress biomarkers showed significant but non-linear and stage-specific differences across TNM categories, with additional differences observed according to selected clinicopathological characteristics; (iii) after Benjamini–Hochberg FDR correction for multiple testing, MDA retained the broadest correlation profile with hematological inflammatory parameters, with significant associations involving NEU, LYM, PLT, RDW, NLR, PLR, MLR, AISI, SII, SIRI, and IIC; (iv) SOD1 retained a more restricted set of significant correlations, involving RDW, NLR, PLR, AISI, SII, and IIC, whereas 8-epi-PGF2α showed no significant correlations after FDR correction; and (v) among the investigated oxidative stress biomarkers, MDA demonstrated the strongest overall prognostic profile, with high time-dependent discriminatory performance for 24-month OS and more modest discrimination for 24-month PFS. In the cutoff-based Cox analyses, high MDA remained significantly associated with shorter PFS after adjustment for age, sex, and either TNM stage or metastatic status. However, when MDA was modeled as a continuous variable per 1-SD increase, the association was significant in univariable analysis but was attenuated and no longer statistically significant after multivariable adjustment, indicating that the estimated prognostic association was sensitive to the modeling of MDA. Collectively, these findings indicate significant differences in systemic oxidative stress according to CRC status and clinicopathological characteristics, predominantly weak-to-moderate statistical correlations between oxidative stress and systemic inflammatory parameters, and a potential prognostic role for MDA that requires external validation [2,20,21].

Oxidative stress is increasingly recognized as a fundamental mechanism involved in colorectal carcinogenesis. Excessive ROS production promotes DNA damage, lipid peroxidation, mitochondrial dysfunction, genomic instability, and activation of oncogenic signaling pathways, thereby contributing to tumor initiation and progression [2,20]. Consistent with these mechanisms, our study demonstrated significantly higher circulating concentrations of 8-epi-PGF2α, MDA, and SOD1 in CRC patients than in controls, indicating differences in systemic oxidative status between the two groups.

Among the investigated biomarkers, 8-epi-PGF2α and MDA represent products of lipid peroxidation, whereas SOD1 reflects activation of endogenous antioxidant defense systems. The concomitant elevation of these markers in CRC patients suggests that increased oxidative lipid damage may coexist with an enhanced antioxidant response. Importantly, the higher circulating SOD1 concentrations observed in CRC patients should not necessarily be interpreted as evidence of a protective or beneficial antioxidant effect. Rather, increased SOD1 may represent an adaptive or compensatory response to increased ROS generation and sustained oxidative stress. In the tumor context, such antioxidant adaptation may enable cells to tolerate elevated oxidative pressure while maintaining redox conditions compatible with survival and proliferation. Previous studies have similarly reported increased concentrations of lipid peroxidation products and altered antioxidant enzyme activity in CRC patients, supporting the involvement of oxidative dysregulation in CRC biology [20,21,22]. In contrast, evidence specifically addressing circulating 8-epi-PGF2α in CRC remains comparatively limited. Accordingly, the significantly higher 8-epi-PGF2α levels observed in our cohort should be interpreted primarily as evidence of increased systemic lipid peroxidation rather than as evidence that 8-epi-PGF2α is an established CRC-specific prognostic biomarker. Our findings further indicate that these alterations are detectable in peripheral blood and may therefore provide complementary systemic information alongside conventional clinicopathological assessment.

8-epi-PGF2α and MDA represent products of lipid peroxidation, whereas SOD1 is an antioxidant enzyme whose circulating concentration may reflect an adaptive or compensatory response to oxidative stress. The concomitant elevation of these markers in CRC patients suggests that increased oxidative lipid damage may coexist with an enhanced antioxidant response. Importantly, the higher circulating SOD1 concentrations observed in CRC patients should not necessarily be interpreted as evidence of a protective or beneficial antioxidant effect. Rather, increased SOD1 may represent an adaptive or compensatory response to increased ROS generation and sustained oxidative stress. In the tumor context, such antioxidant adaptation may enable cells to tolerate elevated oxidative pressure while maintaining redox conditions compatible with survival and proliferation. Previous studies have similarly reported increased concentrations of lipid peroxidation products and altered antioxidant enzyme activity in CRC patients, supporting the involvement of oxidative dysregulation in CRC biology [20,21,22]. Our findings further indicate that these alterations are detectable in peripheral blood and may therefore provide complementary systemic information alongside conventional clinicopathological assessment.

One of the most important findings of our study was MDA’s comparatively strong performance among the oxidative stress biomarkers investigated. MDA is a highly reactive aldehyde generated during the peroxidation of polyunsaturated fatty acids and is widely considered an indicator of oxidative lipid damage [20,21]. Beyond serving as a marker of lipid peroxidation, MDA can form covalent adducts with proteins and nucleic acids, potentially altering cellular function and contributing to genomic instability. Lipid peroxidation may be particularly relevant in CRC because sustained oxidative injury can affect membrane integrity, intracellular signaling, and the tumor microenvironment, biological processes implicated in tumor progression and treatment resistance [20,21]. In our cohort, MDA showed significant differences across several clinicopathological subgroups, significant correlations with multiple hematological inflammatory indices, and prognostic relevance for survival outcomes. Importantly, after accounting for censoring using time-dependent ROC analysis at the predefined 24-month horizon, MDA retained the highest discriminatory performance among the three investigated biomarkers for both endpoints. Its discrimination was particularly high for 24-month OS (AUC = 0.920; bootstrap 95% CI: 0.834–0.978), whereas its performance for 24-month PFS was considerably more modest (AUC = 0.636; bootstrap 95% CI: 0.487–0.773). This distinction is important because it indicates that the prognostic performance of MDA was endpoint-dependent and substantially stronger for 24-month mortality than for disease progression. Nevertheless, the high discrimination observed for 24-month OS should be interpreted cautiously because only 19 deaths occurred during follow-up, all among patients with metastatic disease. Consequently, the observed OS discrimination may be substantially influenced by metastatic status and should be considered exploratory rather than evidence of biomarker discrimination independent of metastatic disease. These observations are consistent with previous investigations reporting elevated MDA concentrations in CRC, particularly in more advanced disease, and suggesting that increased lipid peroxidation may accompany more aggressive tumor biology [21,22].

We observed significant variations in oxidative stress biomarkers across TNM stages. Importantly, these overall differences identified by the Kruskal–Wallis analyses did not follow a strictly linear pattern, as the highest values were generally observed in stage II and stage IV disease rather than showing a progressive increase across all stages. Post hoc analyses further showed that many of the significant pairwise differences involved stages III and IV, although significant differences were also observed between other stage combinations, particularly for MDA. At the level of metastatic status, significant differences between M0 and M1 patients were observed for several oxidative stress biomarkers and inflammatory indices. These findings indicate that systemic redox and inflammatory profiles differed according to metastatic status in our cohort; however, given the observational design, they should not be interpreted as evidence that these circulating biomarkers directly contribute to or mediate metastatic progression. Similar non-linear patterns have occasionally been reported in cancer-related oxidative stress studies and may reflect the complex interactions among tumor burden, host inflammatory responses, metabolic adaptation, and antioxidant compensation mechanisms [2,23]. Nevertheless, these observations should be interpreted with caution, as they may also reflect biological heterogeneity, differences in subgroup sample sizes, or residual confounding rather than a true non-linear relationship between oxidative stress and disease stage. Consequently, these findings should be considered exploratory and require confirmation in larger independent cohorts before definitive biological conclusions can be drawn.

Another notable observation was the unexpected distribution of oxidative stress biomarkers across histological grades. Differences according to histological grade were also non-linear, with several oxidative stress biomarkers showing lower values in G3 tumors than in G1 or G2 tumors. The biological basis of this pattern cannot be established from the present data. Potential explanations may include heterogeneity in metabolic activity, tumor microenvironment composition, immune infiltration, or molecular subtype distribution; alternatively, the observed pattern may partly reflect sampling variability [3,23]. Because the number of patients in individual histological subgroups was limited, these findings should be interpreted with caution and considered exploratory. Larger studies integrating molecular classification and standardized histopathological characterization will be required to determine whether these non-linear patterns reflect reproducible biological differences.

Accumulating evidence indicates that oxidative stress and inflammation are closely interconnected biological processes that mutually reinforce one another during cancer progression [2,3,23]. Reactive oxygen species activate pro-inflammatory transcription factors, including NF-κB and STAT3, whereas inflammatory cells generate additional ROS, establishing a self-sustaining cycle of oxidative and inflammatory signaling. This interaction contributes to tumor proliferation, angiogenesis, epithelial–mesenchymal transition, immune escape, and metastatic dissemination [2,23].

The correlation analysis performed in our cohort provides additional evidence of statistical associations between circulating oxidative stress biomarkers and systemic inflammatory parameters. After Benjamini–Hochberg FDR correction for multiple testing, MDA retained significant positive correlations with neutrophil count, platelet count, RDW, NLR, PLR, MLR, AISI, SII, SIRI, and IIC, together with an inverse correlation with LYM count. In contrast, the nominal correlation between MDA and MCVL did not remain statistically significant after FDR correction. SOD1 showed a more restricted correlation profile, with significant associations retained for RDW, NLR, PLR, AISI, SII, and IIC, whereas 8-epi-PGF2α exhibited no significant correlations with the investigated hematological parameters or inflammatory indices after FDR correction. The heatmap further illustrated the predominance of positive correlation coefficients between MDA and SOD1, as well as several composite inflammatory indices, particularly SII, PLR, AISI, and IIC. Importantly, the significant correlations were predominantly weak to moderate in magnitude and should therefore not be interpreted as evidence of a strong biological relationship. Rather, these patterns indicate that circulating MDA and SOD1 covary statistically with selected systemic inflammatory parameters in CRC. The cross-sectional nature of these analyses precludes conclusions regarding the directionality or causality of these relationships [2,20,23].

The Kaplan–Meier analyses based on the internally derived 24-month thresholds provided additional exploratory information. Significant differences in PFS were observed for 8-epi-PGF2α and MDA, whereas SOD1 did not significantly stratify PFS. These findings should not be considered inconsistent with the time-dependent ROC results, because log-rank testing evaluates differences between entire survival distributions after dichotomization, whereas the time-dependent AUC quantifies individual-level discrimination at a specific prediction horizon. Accordingly, statistically significant separation of Kaplan–Meier curves does not necessarily imply strong discriminatory accuracy at 24 months.

The prognostic performance of MDA varied depending on the survival outcome evaluated. In the 24-month time-dependent ROC analysis accounting for censored observations, MDA demonstrated high discriminatory performance for OS (AUC = 0.920; bootstrap 95% CI: 0.834–0.978), whereas its discriminatory performance for PFS was considerably more modest (AUC = 0.636; bootstrap 95% CI: 0.487–0.773). Thus, the strong discrimination observed for 24-month OS should not be extrapolated to PFS. The Cox proportional hazards regression analyses provided complementary prognostic information on PFS. When MDA was dichotomized using the internally derived 24-month time-dependent ROC cutoff of ≥552.89 ng/mL, high MDA remained significantly associated with an increased risk of progression in both multivariable models, regardless of whether adjustment was made for TNM stage or metastatic status. However, the sensitivity analysis modeling MDA as a continuous variable provided a more nuanced result. Although each 1-SD increase in MDA (255.19 ng/mL) was significantly associated with a higher hazard of progression in univariable analysis (HR = 1.51, 95% CI: 1.20–1.88, *p* < 0.001), this association was attenuated and was no longer statistically significant after adjustment for age, sex, and TNM stage (HR = 1.18, 95% CI: 0.90–1.53, *p* = 0.234) or for age, sex, and metastatic status (HR = 1.22, 95% CI: 0.92–1.63, *p* = 0.172). The difference between the cutoff-based and continuous-variable analyses indicates that the estimated prognostic association of MDA with PFS is sensitive to its modeling and warrants cautious interpretation. Importantly, the modest time-dependent discriminatory performance of MDA for 24-month PFS, the significant Kaplan–Meier separation, and the Cox regression results represent distinct aspects of prognostic performance. The time-dependent AUC assesses individual-level discrimination at the predefined 24-month prediction horizon while accounting for censoring, whereas Cox regression evaluates the association between MDA and the hazard of progression over the follow-up period. Taken together, these findings support a potential prognostic association between circulating MDA and PFS but do not establish MDA as an independently validated prognostic biomarker. Given the observational design, they also cannot establish that MDA itself causally contributes to disease progression. The observed associations are biologically plausible given the established involvement of lipid peroxidation and oxidative stress in tumor progression, therapeutic resistance, and adverse clinical outcomes in CRC [21,23]. However, because the MDA cutoff was internally derived and evaluated within the same cohort, the continuous association was not statistically significant after multivariable adjustment, and the number of PFS events was limited, the prognostic relevance of MDA should be considered exploratory and requires validation in larger independent external cohorts before clinical implementation.

The use of PFS rather than DFS reflects the inclusion of patients with metastatic disease in the study cohort and provides a clinically appropriate endpoint for evaluating disease progression across the entire CRC population.

A particular strength of the present study is the inclusion of two recently proposed inflammatory indices, MCVL and IIC. While conventional indices such as NLR, PLR, and SII have been extensively investigated in CRC, evidence on MCVL, and especially IIC, remains limited. A recent study by Șerban et al. reported potential diagnostic and prognostic relevance of MCVL and IIC in colorectal adenocarcinoma [14]. In the present cohort, both indices showed correlations with oxidative stress biomarkers, suggesting that they may reflect components of the systemic oxidative–inflammatory profile. However, these findings should be considered exploratory. Neither MCVL nor IIC can currently be regarded as clinically validated prognostic biomarkers, and their incremental prognostic value beyond established inflammatory indices and clinicopathological factors remains to be demonstrated in independent cohorts.

### 4.1. Clinical Implications, Future Directions

From a clinical perspective, the present findings suggest that circulating oxidative stress biomarkers, particularly MDA, warrant further investigation as adjunctive markers for CRC risk stratification. MDA demonstrated high time-dependent discrimination for 24-month OS but only modest discrimination for 24-month PFS. In the Cox analyses, MDA dichotomized according to the internally derived 24-month time-dependent ROC cutoff remained significantly associated with PFS after multivariable adjustment; however, this association was attenuated and no longer statistically significant when MDA was modeled continuously per 1-SD increase. This model-dependent difference indicates that the prognostic relevance of MDA remains exploratory and is insufficient to support its clinical implementation at present. Moreover, the observed correlations between oxidative stress biomarkers and composite inflammatory indices support further investigation of their combined assessment as a potential approach to characterizing the systemic oxidative–inflammatory profile in CRC [2,21,23].

Future studies should validate these findings in larger, independent multicenter cohorts and integrate oxidative stress biomarkers with molecular, genomic, and immune profiling approaches. Particular attention should be given to externally validating both the prognostic performance and the optimal cutoff of MDA, as well as the association between continuously modeled MDA concentrations and clinical outcomes. Future studies should also investigate the potential clinical utility of emerging inflammatory indices such as MCVL and IIC. Prospective longitudinal studies assessing dynamic changes in oxidative stress biomarkers during treatment may further determine whether these markers provide clinically meaningful information regarding therapeutic response, disease progression, and long-term outcomes in patients with CRC.

### 4.2. Limitations

Several limitations should be acknowledged. First, although the study had a prospective observational cohort design with longitudinal follow-up for survival outcomes, oxidative stress biomarkers and hematological inflammatory indices were assessed at a single pretreatment time point. Therefore, the cross-sectional nature of the baseline biomarker analyses precludes causal or directional inference regarding the relationships between oxidative stress, systemic inflammation, and clinicopathological characteristics. Second, this was a single-center study with a relatively modest sample size, particularly within certain TNM and histological subgroups. The relatively small control group (n = 40), recruited from individuals undergoing routine investigations within the same institutional setting, may also limit the representativeness of the reference population and should be considered when interpreting the between-group comparisons. Because no formal a priori sample size calculation was performed, the study may have been underpowered for some subgroup and survival analyses, particularly those involving relatively small numbers of participants or events. Furthermore, the large number of biomarkers, inflammatory indices, clinicopathological subgroup comparisons, and correlation analyses increases the risk of type I error due to multiple testing, despite the use of Bonferroni correction for post hoc comparisons. Therefore, findings from the broader secondary and exploratory analyses should be interpreted cautiously as hypothesis-generating and require confirmation in independent cohorts. Third, oxidative stress biomarkers were measured only in serum, without parallel assessment of tumor tissue expression. Furthermore, ELISA measurements were performed as single determinations rather than in duplicate due to limited sample volume and study resources. Although all samples were analyzed using the same standardized protocol and the manufacturer-reported intra- and inter-assay coefficients of variation were within the specified analytical ranges, the absence of duplicate measurements may have increased the influence of analytical variability and should be considered a methodological limitation. Another limitation relates to the use of 24-month time-dependent ROC-derived cutoff values, which were both derived and evaluated within the same study cohort. Consequently, these thresholds should be considered exploratory and hypothesis-generating rather than definitive clinical decision limits. External validation in independent and larger prospective cohorts will be necessary to confirm their reproducibility and clinical applicability. Furthermore, the prognostic association between MDA and PFS was sensitive to the method used to model the biomarker. Although dichotomized MDA based on the internally derived 24-month time-dependent ROC cutoff remained significantly associated with PFS in the multivariable Cox models, the corresponding associations were attenuated and no longer statistically significant when MDA was modeled continuously per 1-SD increase. This discrepancy limits the robustness of the cutoff-based findings and further supports their interpretation as exploratory rather than as evidence of an independently validated prognostic effect. In addition, detailed treatment-related variables, including surgical procedures, neoadjuvant treatment, and systemic therapy for metastatic disease, were not available in a standardized manner and therefore could not be incorporated into the analyses. Because treatment exposure may influence both survival outcomes and circulating inflammatory profiles, the inability to adjust for these variables may introduce residual confounding, particularly in prognostic analyses. Finally, the relatively limited number of survival events—19 deaths for OS and 39 events for PFS—may have reduced the statistical power of the survival analyses. In particular, the limited number of OS events and their occurrence exclusively among patients with metastatic disease precluded reliable multivariable Cox regression for OS because of the risk of model instability and overfitting. Consequently, the high time-dependent discriminatory performance observed for MDA in the 24-month OS analysis may be substantially influenced by metastatic status and should be regarded as exploratory rather than as evidence of mortality discrimination independent of metastatic disease.

## 5. Conclusions

In conclusion, CRC patients exhibited significantly higher serum levels of 8-epi-PGF2α, MDA, and SOD1 than healthy controls, indicating differences in systemic oxidative status. Among the oxidative stress biomarkers investigated, MDA showed the most consistent associations with clinicopathological characteristics, systemic inflammatory parameters, and survival outcomes in the present cohort. After correction for multiple testing, MDA and, to a lesser extent, SOD1 retained significant correlations with several hematological inflammatory indices, although these associations were predominantly weak to moderate and should not be interpreted as evidence of causal biological relationships. Prognostic analyses further identified MDA as the biomarker with the highest discriminatory performance for 24-month OS, whereas its performance for 24-month PFS was substantially more modest. High MDA, defined using the internally derived 24-month time-dependent ROC threshold, remained significantly associated with shorter PFS after adjustment for age, sex, and either TNM stage or metastatic status. However, this association was attenuated and no longer statistically significant after multivariable adjustment when MDA was modeled continuously, indicating that its prognostic association with PFS is sensitive to the analytical approach. Moreover, the limited number of OS events and their occurrence exclusively among patients with metastatic disease require cautious interpretation of the apparently high discrimination observed for OS. Overall, these findings support MDA as a promising candidate marker that reflects the oxidative–inflammatory profile and potentially provides prognostic information in CRC, rather than as an independently validated prognostic biomarker. The non-linear patterns observed across clinicopathological subgroups, the exploratory nature of several analyses, and the internally derived prognostic thresholds further emphasize the need for validation. Larger multicenter prospective studies incorporating standardized treatment data and independent validation cohorts are required to determine whether MDA and related oxidative–inflammatory biomarkers provide clinically meaningful information beyond established CRC prognostic factors.

## Figures and Tables

**Table 1:** Patient demographic and clinical characteristics.

Characteristics	CRC Group (*n* = 140)	Control Group (*n* = 40)	*p*-Value
Age (yrs) (mean ± SD)	65.66 ± 7.60	61.75 ± 13.92	0.095
Gender, Female/Male (*n*)	56/84	18/22	0.571
Area of residence, Rural/Urban (*n*)	79/61	22/18	-
Tumor extension (T) (*n*)			
T1	6	NA	-
T2	8	NA	-
T3	89	NA	-
T4	37	NA	-
Regional lymph node metastasis (N) (*n*)			
N0	39	NA	-
N1	66	NA	-
N2	35	NA	-
Distant metastasis (M) (*n*)			
M0	97	NA	-
M1	43	NA	-
TNM Stage of WHO Classification of Tumors 2019 (*n*)			
I	14	NA	-
II	23	NA	-
III	60	NA	-
IV	43	NA	-
Tumor Grade (G) (*n*)			
G1	44	NA	-
G2	41	NA	-
G3	55	NA	-
Tumor side (*n*)			
Right	42	NA	-
Left	98	NA	-

**Table 2:** Median values and statistical significance of oxidative stress biomarkers, hematological parameters, and inflammatory indices in CRC patients and controls.

Parameter Mediană [IQR]	CRC Group (*n* = 140)	Control Group (*n* = 40)	*p*-Value
8-epi-PGF2α (pg/mL)	381.91 [228.54–584.68]	270.64 [234.83–374.77]	0.016
MDA (ng/mL)	331.05 [242.23–447.26]	92.43 [70.34–115.90]	<0.001
SOD1 (pg/mL)	2993.47 [1741.87–4385.84]	1037.87 [590.14–1761.20]	<0.001
Hb (g/dL)	10.56 [8.10–12.44]	13.30 [12.60–14.20]	<0.001
WBC (×10^3^/μL)	8.35 [6.84–9.98]	6.61 [5.26–8.40]	<0.001
NEU (×10^3^/μL)	5.13 [4.07–6.67]	3.90 [2.81–5.93]	0.004
LYM (×10^3^/μL)	2.11 [1.57–2.78]	1.48 [1.16–2.12]	0.010
MON (×10^3^/μL)	0.59 [0.44–0.71]	0.59 [0.42–0.88]	0.113
PLT (×10^3^/μL)	267.0 [223.9–328.5]	250.0 [214.0–288.0]	0.198
MCV (fL)	89.56 [83.92–94.38]	89.95 [86.90–93.83]	0.881
MCH (pg)	29.29 [27.05–30.63]	29.05 [27.78–30.28]	0.869
MCHC (g/dL)	32.30 [31.28–33.14]	32.50 [32.05–33.03]	0.463
RDW (%)	13.29 [12.50–15.23]	13.80 [13.03–15.20]	0.155
NLR	2.36 [1.72–3.69]	2.08 [1.37–2.97]	0.454
PLR	127.27 [94.54–177.22]	130.57 [95.95–180.69]	0.135
MLR	0.266 [0.196–0.355]	0.402 [0.281–0.573]	<0.001
AISI	330.87 [207.43–573.77]	337.49 [174.62–799.83]	0.873
SII	608.45 [426.22–1042.40]	515.96 [273.79–745.70]	0.206
SIRI	1.31 [0.88–2.07]	1.38 [0.72–3.00]	0.658
MCVL	40.82 [31.06–59.39]	59.93 [46.74–60.33]	0.037
IIC	2.92 [1.96–4.83]	2.77 [1.51–3.41]	0.418

**Table 3:** Comparison of TNM stage and biomarkers in the study group.

Parameter Mediană [IQR]	TNM Stage I (*n* = 14)	TNM Stage II (*n* = 23)	TNM Stage III (*n* = 60)	TNM Stage IV (*n* = 43)	*p*-Value
8-epi-PGF2α (pg/mL)	410.60 [375.75–440.40]	267.15 [200.88–473.31]	255.01 [174.86–382.57]	413.91 [391.11–456.95]	<0.001
MDA (ng/mL)	331.05 [318.46–459.40]	600.00 [343.36–619.88]	244.53 [231.25–276.79]	538.95 [489.73–612.78]	<0.001
SOD1 (pg/mL)	3385.62 [2980.39–3522.88]	3398.69 [1819.15–3898.69]	1973.99 [1607.96–2467.58]	3398.69 [3215.68–3712.41]	<0.001
Hb (g/dL)	9.25 [8.70–10.91]	10.77 [8.16–13.12]	11.27 [8.54–12.90]	9.20 [7.80–12.03]	0.195
WBC (×10^3^/μL)	6.80 [6.73–8.61]	9.32 [8.30–10.50]	8.22 [6.89–10.08]	8.40 [6.71–9.95]	0.087
NEU (×10^3^/μL)	4.66 [3.91–5.41]	5.84 [5.30–6.86]	4.83 [3.69–6.39]	5.50 [4.68–6.80]	0.068
LYM (×10^3^/μL)	1.69 [1.29–2.34]	2.48 [1.90–3.02]	2.60 [1.68–3.24]	1.90 [1.31–2.38]	<0.001
MON (×10^3^/μL)	0.62 [0.40–0.69]	0.61 [0.55–0.73]	0.54 [0.40–0.70]	0.60 [0.49–0.80]	0.153
PLT (×10^3^/μL)	307.60 [289.57–390.25]	297.30 [246.50–355.00]	250.40 [215.78–317.48]	243.60 [204.65–321.20]	0.084
MCV (fL)	86.68 [79.27–92.97]	90.70 [84.83–93.55]	91.18 [86.87–94.21]	87.60 [76.26–95.29]	0.402
MCH (pg)	28.15 [24.54–30.39]	29.30 [27.45–30.80]	29.59 [28.16–30.75]	28.60 [23.25–30.62]	0.280
MCHC (g/dL)	32.27 [31.20–32.53]	32.20 [31.26–33.15]	32.65 [31.61–33.43]	31.98 [31.01–32.94]	0.179
RDW (%)	13.89 [12.21–18.37]	13.21 [12.69–15.02]	13.04 [12.30–14.12]	13.90 [13.04–17.46]	0.010
NLR	2.50 [1.62–3.98]	2.24 [1.86–3.10]	1.94 [1.44–2.84]	2.97 [2.21–4.21]	0.002
PLR	152.44 [131.60–290.03]	132.06 [89.61–173.40]	107.08 [72.22–128.81]	135.19 [115.33–214.11]	<0.001
MLR	0.32 [0.17–0.50]	0.30 [0.22–0.37]	0.22 [0.16–0.34]	0.30 [0.24–0.48]	0.006
AISI	264.45 [195.60–991.25]	642.23 [312.07–877.16]	265.88 [187.89–396.32]	495.38 [296.95–737.70]	0.001
SII	735.06 [489.01–1387.98]	724.53 [535.87–1098.39]	503.66 [369.70–713.85]	854.11 [536.25–1154.93]	0.002
SIRI	1.15 [0.68–2.68]	2.04 [1.18–2.73]	1.03 [0.67–1.88]	1.91 [1.15–2.75]	0.003
MCVL	52.70 [40.52–63.45]	34.93 [31.46–46.87]	37.71 [26.62–47.01]	47.42 [35.50–68.75]	0.006
IIC	3.37 [1.88–5.87]	3.23 [2.29–4.39]	2.23 [1.72–3.31]	3.34 [2.53–6.18]	0.001

**Table 4:** Significant Pairwise Comparisons after Bonferroni Correction.

Variable	Significant Comparison	Adjusted *p*-Value
8-epi-PGF2α	TNM III vs. TNM IV	<0.001
MDA	TNM I vs. TNM III	<0.001
MDA	TNM I vs. TNM IV	<0.001
MDA	TNM II vs. TNM III	<0.001
MDA	TNM III vs. TNM IV	<0.001
SOD1	TNM III vs. TNM IV	<0.001
LYM	TNM II vs. TNM IV	0.015
LYM	TNM III vs. TNM IV	0.001
RDW	TNM III vs. TNM IV	0.005
NLR	TNM III vs. TNM IV	0.001
PLR	TNM I vs. TNM III	0.023
PLR	TNM III vs. TNM IV	<0.001
MLR	TNM III vs. TNM IV	0.004
AISI	TNM II vs. TNM III	0.012
AISI	TNM III vs. TNM IV	0.003
SII	TNM II vs. TNM III	0.041
SII	TNM III vs. TNM IV	0.005
SIRI	TNM III vs. TNM IV	0.003
MCVL	TNM III vs. TNM IV	0.016
IIC	TNM III vs. TNM IV	0.001

**Table 5:** Comparison of oxidative stress biomarkers, hematological parameters, and inflammatory indices according to T stage.

Variable	T1 + T2 (*n* = 14)	T3 (*n* = 89)	T4 (*n* = 37)	*p*-Value
8-epi-PGF2α (pg/mL)	399.01 [233.67–440.40]	380.79 [234.83–443.27]	400.66 [226.56–460.26]	0.807
MDA (ng/mL)	323.09 [276.66–355.18]	322.03 [242.62–511.05]	553.71 [239.84–614.41]	0.076
SOD1 (pg/mL)	2980.39 [1670.47–3503.27]	3137.25 [1747.23–3686.27]	2127.66 [1702.13–3398.69]	0.452
Hb (g/dL)	10.27 [8.92–11.93]	10.37 [7.76–12.06]	11.59 [8.80–13.40]	0.045
WBC (×10^3^/μL)	7.12 [6.75–8.31]	8.40 [6.74–10.46]	8.53 [7.70–9.89]	0.145
NEU (×10^3^/μL)	4.58 [4.48–4.94]	5.40 [4.07–6.80]	5.01 [4.07–6.33]	0.293
LYM (×10^3^/μL)	1.79 [1.50–2.34]	2.00 [1.50–2.70]	2.60 [2.02–3.04]	0.017
MON (×10^3^/μL)	0.62 [0.42–0.69]	0.60 [0.46–0.73]	0.50 [0.40–0.68]	0.291
PLT (×10^3^/μL)	302.45 [234.75–367.48]	253.00 [213.40–318.90]	272.00 [226.00–326.90]	0.378
MCV (fL)	87.15 [82.20–90.45]	90.40 [84.00–94.30]	89.00 [82.69–95.71]	0.541
MCH (pg)	28.60 [26.62–29.66]	29.40 [27.12–30.60]	29.40 [27.05–30.99]	0.660
MCHC (g/dL)	32.46 [31.72–32.87]	32.30 [31.30–33.20]	32.10 [31.16–33.00]	0.767
RDW (%)	12.34 [12.11–16.40]	13.50 [12.80–15.20]	13.10 [12.53–15.20]	0.418
NLR	2.35 [1.73–3.58]	2.64 [1.76–3.54]	1.98 [1.45–2.92]	0.090
PLR	141.33 [131.20–193.55]	127.62 [94.59–178.17]	107.53 [90.77–127.31]	0.025
MLR	0.29 [0.18–0.42]	0.29 [0.22–0.39]	0.21 [0.15–0.29]	0.006
AISI	303.68 [200.54–662.95]	392.41 [232.76–744.27]	295.21 [166.25–477.97]	0.104
SII	662.28 [489.01–1119.44]	656.12 [432.78–1015.53]	536.25 [372.15–898.57]	0.225
SIRI	1.26 [0.85–2.35]	1.60 [0.94–2.53]	1.08 [0.62–2.00]	0.065
MCVL	49.37 [38.29–60.44]	43.58 [31.47–60.10]	35.92 [27.27–44.97]	0.030
IIC	2.88 [1.98–4.54]	3.14 [2.10–4.65]	2.39 [1.73–3.28]	0.097

**Table 6:** Comparison of oxidative stress biomarkers, hematological parameters, and inflammatory indices according to N stage.

Variable	N0 (*n* = 39)	N1 (*n* = 66)	N2 (*n* = 35)	*p*-Value
8-epi-PGF2α (pg/mL)	380.79 [232.04–457.58]	373.17 [226.56–413.91]	430.46 [280.24–474.58]	0.145
MDA (ng/mL)	486.45 [321.29–606.02]	283.28 [240.14–463.27]	479.06 [237.11–612.78]	0.002
SOD1 (pg/mL)	3398.69 [1835.11–3607.85]	2095.74 [1707.45–3450.98]	2218.21 [1740.83–3411.77]	0.243
Hb (g/dL)	10.54 [8.62–11.87]	10.42 [7.80–12.80]	11.15 [8.64–12.88]	0.450
WBC (×10^3^/μL)	8.72 [7.06–10.55]	8.13 [6.80–9.89]	8.40 [6.88–9.87]	0.431
NEU (×10^3^/μL)	5.59 [4.58–6.76]	4.98 [3.94–6.46]	5.40 [4.03–6.51]	0.217
LYM (×10^3^/μL)	2.34 [1.52–2.70]	2.07 [1.42–2.98]	2.03 [1.83–2.62]	0.975
MON (×10^3^/μL)	0.64 [0.54–0.76]	0.56 [0.46–0.73]	0.50 [0.39–0.69]	0.131
PLT (×10^3^/μL)	297.30 [236.50–373.70]	247.20 [201.28–320.02]	270.00 [234.00–316.25]	0.047
MCV (fL)	89.00 [83.93–94.56]	90.33 [84.59–94.19]	87.75 [82.39–95.30]	0.935
MCH (pg)	29.21 [27.06–30.65]	29.40 [27.57–30.58]	29.08 [25.85–30.80]	0.964
MCHC (g/dL)	32.40 [31.30–33.02]	32.30 [31.43–33.18]	32.10 [31.01–33.32]	0.984
RDW (%)	13.21 [12.50–14.52]	13.49 [12.58–15.20]	13.27 [12.45–16.00]	0.935
NLR	2.45 [1.84–3.85]	2.49 [1.50–3.48]	2.22 [1.80–3.10]	0.426
PLR	132.06 [105.44–190.09]	123.49 [86.80–167.83]	121.61 [98.05–151.09]	0.211
MLR	0.31 [0.23–0.40]	0.26 [0.20–0.39]	0.22 [0.19–0.33]	0.146
AISI	541.93 [257.73–898.31]	313.40 [193.66–605.55]	295.21 [219.09–482.55]	0.049
SII	731.94 [489.01–1214.21]	586.64 [370.07–996.17]	575.66 [423.71–859.30]	0.083
SIRI	1.93 [1.11–2.68]	1.27 [0.86–2.15]	1.13 [0.84–2.07]	0.144
MCVL	39.31 [31.93–57.23]	43.77 [28.94–60.10]	40.39 [29.66–49.55]	0.796
IIC	3.23 [2.10–4.69]	2.99 [1.89–3.91]	2.54 [1.97–3.75]	0.404

**Table 7:** Comparison of oxidative stress biomarkers, hematological parameters, and inflammatory indices according to metastatic status.

Variable	M0 (*n* = 97)	M1 (*n* = 43)	*p*-Value
8-epi-PGF2α (pg/mL)	260.37 [178.70–443.27]	413.91 [391.11–456.95]	<0.001
MDA (ng/mL)	263.67 [232.69–344.92]	538.95 [489.73–612.78]	<0.001
SOD1 (pg/mL)	1946.81 [1648.69–3503.27]	3398.69 [3215.68–3712.41]	<0.001
Hb (g/dL)	10.96 [8.63–12.57]	9.20 [7.80–12.03]	0.117
WBC (×10^3^/μL)	8.32 [6.90–9.97]	8.40 [6.71–9.95]	0.663
NEU (×10^3^/μL)	5.01 [3.95–6.58]	5.50 [4.68–6.80]	0.251
LYM (×10^3^/μL)	2.40 [1.68–3.08]	1.90 [1.31–2.38]	<0.001
MON (×10^3^/μL)	0.58 [0.43–0.70]	0.60 [0.49–0.80]	0.367
PLT (×10^3^/μL)	267.60 [224.60–323.00]	243.60 [204.65–321.20]	0.406
MCV (fL)	90.49 [86.35–94.30]	87.60 [76.26–95.29]	0.238
MCH (pg)	29.40 [28.00–30.60]	28.60 [23.25–30.62]	0.131
MCHC (g/dL)	32.47 [31.40–33.20]	31.98 [31.01–32.94]	0.064
RDW (%)	13.10 [12.33–14.30]	13.90 [13.04–17.46]	0.002
NLR	2.03 [1.59–3.04]	2.97 [2.21–4.21]	<0.001
PLR	120.17 [84.77–154.28]	135.19 [115.33–214.11]	0.002
MLR	0.25 [0.18–0.37]	0.30 [0.24–0.48]	0.006
AISI	292.94 [195.26–707.63]	495.38 [296.95–737.70]	0.011
SII	575.66 [396.34–809.19]	854.11 [536.25–1154.93]	0.009
SIRI	1.13 [0.84–2.04]	1.91 [1.15–2.75]	0.006
MCVL	38.68 [28.17–51.69]	47.42 [35.50–68.75]	0.004
IIC	2.73 [1.89–3.58]	3.34 [2.53–6.18]	0.001

**Table 8:** Comparison of Oxidative Stress Biomarkers and Inflammatory Indices According to Tumor Grade.

Variable	G1 (*n* = 44)	G2 (*n* = 41)	G3 (*n* = 55)	*p*-Value
8-epi-PGF2α (pg/mL)	376.48 [228.57–447.02]	382.57 [242.52–413.91]	382.57 [208.77–484.81]	0.894
MDA (ng/mL)	446.25 [263.57–576.20]	463.48 [267.92–560.27]	256.64 [231.60–479.47]	0.015
SOD1 (pg/mL)	3202.61 [1747.34–3732.02]	3346.41 [1946.81–3738.56]	1884.36 [1622.99–3411.77]	0.005
Hb (g/dL)	10.14 [7.96–11.36]	10.83 [8.59–12.80]	11.20 [8.42–12.82]	0.273
WBC (×10^3^/μL)	9.18 [6.88–10.63]	8.37 [6.97–9.97]	8.10 [6.70–9.43]	0.113
NEU (×10^3^/μL)	5.59 [4.54–7.18]	5.79 [4.62–7.09]	4.74 [3.58–5.54]	0.014
LYM (×10^3^/μL)	2.24 [1.68–2.70]	1.92 [1.40–2.60]	2.39 [1.63–3.20]	0.033
MON (×10^3^/μL)	0.64 [0.51–0.78]	0.60 [0.47–0.80]	0.51 [0.38–0.64]	0.010
PLT (×10^3^/μL)	276.90 [231.25–327.45]	272.00 [209.30–344.20]	251.80 [222.60–312.25]	0.433
MCV (fL)	88.33 [81.86–94.30]	89.00 [82.69–94.20]	91.80 [86.50–95.05]	0.177
MCH (pg)	28.84 [26.20–30.59]	28.90 [25.90–30.50]	29.60 [28.24–30.65]	0.193
MCHC (g/dL)	32.19 [31.20–32.99]	32.10 [31.50–33.00]	32.56 [31.36–33.45]	0.267
RDW (%)	13.16 [12.20–16.18]	14.40 [12.99–17.42]	13.10 [12.46–13.95]	0.007
NLR	2.54 [1.96–3.96]	2.84 [1.96–3.76]	1.93 [1.46–2.76]	0.001
PLR	131.45 [86.30–178.17]	133.68 [119.67–241.34]	107.53 [88.17–133.44]	0.010
MLR	0.29 [0.21–0.40]	0.31 [0.25–0.47]	0.22 [0.16–0.31]	<0.001
AISI	498.95 [254.47–780.15]	477.97 [310.57–978.63]	245.84 [187.14–382.28]	<0.001
SII	716.08 [469.53–1212.67]	854.11 [582.65–1172.50]	504.94 [374.38–638.28]	<0.001
SIRI	1.85 [1.11–2.64]	1.92 [1.21–3.21]	0.98 [0.67–1.40]	<0.001
MCVL	38.15 [31.66–49.54]	45.16 [36.15–60.10]	37.37 [27.67–53.83]	0.058
IIC	3.07 [2.10–4.88]	3.30 [2.73–5.84]	2.27 [1.78–3.22]	<0.001

**Table 9:** Significant Pairwise Mann–Whitney U Comparisons According to Tumor Grade after Bonferroni Correction.

Variable	Significant Comparison	Adjusted *p*-Value
MDA	G2 vs. G3	0.009
SOD1	G1 vs. G3	0.011
SOD1	G2 vs. G3	0.004
NEU	G2 vs. G3	0.036
NLR	G1 vs. G3	0.006
NLR	G2 vs. G3	<0.001
PLR	G2 vs. G3	0.028
MLR	G1 vs. G3	0.009
MLR	G2 vs. G3	<0.001
AISI	G1 vs. G3	0.003
AISI	G2 vs. G3	<0.001
SII	G1 vs. G3	0.007
SII	G2 vs. G3	<0.001
SIRI	G1 vs. G3	0.002
SIRI	G2 vs. G3	<0.001
IIC	G1 vs. G3	0.012
IIC	G2 vs. G3	<0.001

**Table 10:** Tumor location/sidedness analysis.

Variable	Right (*n* = 42)	Left (*n* = 98)	*p*-Value
8-epi-PGF2α (pg/mL)	386.65 [230.71–440.07]	381.25 [226.56–464.87]	0.754
MDA (ng/mL)	476.84 [256.75–607.71]	315.62 [238.77–508.89]	0.094
SOD1 (pg/mL)	3398.70 [1957.45–3777.78]	2095.74 [1715.43–3424.84]	0.012
Hb (g/dL)	10.82 [7.97–12.56]	10.54 [8.13–12.41]	0.875
WBC (×10^3^/μL)	8.35 [6.75–9.85]	8.35 [6.94–9.99]	0.674
NEU (×10^3^/μL)	5.22 [3.81–6.81]	5.11 [4.28–6.64]	0.730
LYM (×10^3^/μL)	2.08 [1.44–2.98]	2.11 [1.60–2.75]	0.913
MON (×10^3^/μL)	0.57 [0.36–0.76]	0.59 [0.46–0.71]	0.249
PLT (×10^3^/μL)	283.80 [231.50–339.90]	256.80 [221.80–319.17]	0.195
MCV (fL)	87.67 [78.61–94.28]	90.63 [86.31–94.90]	0.067
MCH (pg)	28.63 [24.10–30.53]	29.45 [27.84–30.68]	0.105
MCHC (g/dL)	31.93 [30.81–33.00]	32.40 [31.56–33.18]	0.199
RDW (%)	14.45 [13.07–17.25]	13.10 [12.34–14.10]	<0.001
NLR	2.21 [1.75–3.53]	2.46 [1.64–3.46]	0.879
PLR	126.02 [101.48–186.43]	127.28 [92.92–169.04]	0.362
MLR	0.26 [0.19–0.42]	0.27 [0.20–0.37]	0.940
AISI	380.26 [216.57–661.45]	315.95 [203.14–739.73]	0.919
SII	619.84 [479.33–991.58]	599.72 [406.94–1000.56]	0.538
SIRI	1.40 [0.80–2.20]	1.25 [0.91–2.39]	0.801
MCVL	38.36 [27.49–63.12]	41.82 [31.83–52.63]	0.708
IIC	3.17 [2.29–4.75]	2.91 [1.90–4.13]	0.336

**Table 11:** Time-dependent ROC analysis of oxidative stress biomarkers for 24-month overall survival and progression-free survival with internal bootstrap validation.

Endpoint	Marker	Cut-Off	AUC at 24 Months	Bootstrap 95% CI	Sensitivity (%)	Specificity (%)
OS	8-epi-PGF2α	309.07 pg/mL	0.564	0.400–0.722	88.3	38.9
OS	MDA	538.95 ng/mL	0.920	0.834–0.978	100.0	75.0
OS	SOD1	3241.83 pg/mL	0.665	0.501–0.805	75.4	61.1
PFS	8-epi-PGF2α	430.46 pg/mL	0.548	0.395–0.679	49.0	64.3
PFS	MDA	552.89 ng/mL	0.636	0.487–0.773	56.9	76.2
PFS	SOD1	1648.69 pg/mL	0.423	0.289–0.549	90.3	16.7

Note: AUC, area under the curve; CI, confidence interval; OS, overall survival; PFS, progression-free survival. Time-dependent AUCs were estimated over a predefined 24-month prediction horizon using a cumulative/dynamic ROC approach that accounts for censoring. The 95% CIs were obtained using 1000 bootstrap resamples. Cutoff values were internally derived using the time-dependent Youden index and should be considered exploratory.

**Table 12:** Cox proportional hazards regression analyses evaluating the association between serum MDA levels and progression-free survival (PFS).

Variable	HR	95% CI	*p*-Value
*PFS Cox Model 1: cutoff-based MDA, adjusted for age, sex, and TNM stage*			
High MDA	2.49	1.29–4.80	0.006
Age	1.02	0.97–1.08	0.470
Male sex	0.59	0.29–1.17	0.131
TNM stage	2.50	1.52–4.13	<0.001
*PFS Cox Model 2: cutoff-based MDA, adjusted for age, sex, and metastatic status*			
High MDA	2.40	1.22–4.72	0.011
Age	1.03	0.98–1.09	0.273
Male sex	0.49	0.24–0.98	0.044
Metastatic status M1	2.46	1.21–4.98	0.013
*Continuous MDA sensitivity analysis*			
MDA per 1-SD increase, univariable	1.51	1.20–1.88	<0.001
MDA per 1-SD increase, adjusted for age, sex, and TNM stage	1.18	0.90–1.53	0.234
MDA per 1-SD increase, adjusted for age, sex, and metastatic status	1.22	0.92–1.63	0.172

High MDA was defined using the internally derived 24-month time-dependent ROC cutoff value of ≥552.89 ng/mL. In the continuous-variable sensitivity analysis, MDA was standardized, and HRs are reported per 1-SD increase (255.19 ng/mL). Cutoff-based Model 1 was adjusted for age, sex, and TNM stage, whereas cutoff-based Model 2 was adjusted for age, sex, and metastatic status. The same covariate structures were used for the corresponding continuous-MDA sensitivity analyses. TNM stage and metastatic status were entered into separate multivariable models to avoid collinearity. Hazard ratios (HRs) are presented with 95% confidence intervals (CIs). The proportional hazards assumption was assessed using Schoenfeld residuals and was not significantly violated in either multivariable model (all *p* > 0.05).

**Table 13:** Spearman correlations between oxidative stress biomarkers and hematological indices.

Hematological Index	8-epi-PGF2α ρ (*p*; *q*)	MDA ρ (*p*; *q*)	SOD1 ρ (*p*; *q*)
Hb	−0.160 (0.058; 0.116)	−0.130 (0.127; 0.208)	−0.048 (0.571; 0.642)
WBC	0.035 (0.678; 0.720)	0.070 (0.408; 0.489)	0.072 (0.401; 0.489)
NEU	0.078 (0.359; 0.462)	0.210 (0.013; 0.041)	0.179 (0.035; 0.079)
LYM	−0.114 (0.179; 0.268)	−0.209 (0.013; 0.041)	−0.190 (0.025; 0.061)
MON	0.107 (0.209; 0.290)	0.105 (0.219; 0.296)	−0.021 (0.808; 0.824)
PLT	−0.018 (0.829; 0.829)	0.215 (0.011; 0.040)	0.196 (0.020; 0.054)
MCV	−0.061 (0.476; 0.551)	−0.087 (0.307; 0.405)	−0.183 (0.030; 0.068)
MCH	−0.025 (0.767; 0.798)	−0.092 (0.279; 0.377)	−0.186 (0.028; 0.066)
MCHC	−0.010 (0.904; 0.904)	−0.066 (0.441; 0.519)	−0.143 (0.092; 0.158)
RDW	0.038 (0.656; 0.709)	0.220 (0.009; 0.035)	0.300 (<0.001; <0.005)
NLR	0.139 (0.101; 0.168)	0.277 (<0.001; <0.005)	0.270 (0.001; 0.005)
PLR	0.079 (0.354; 0.462)	0.326 (<0.001; <0.005)	0.304 (<0.001; <0.005)
MLR	0.118 (0.166; 0.256)	0.230 (0.006; 0.025)	0.147 (0.083; 0.149)
AISI	0.138 (0.103; 0.168)	0.319 (<0.001; <0.005)	0.239 (0.004; 0.020)
SII	0.085 (0.317; 0.410)	0.336 (<0.001; <0.005)	0.350 (<0.001; <0.005)
SIRI	0.146 (0.084; 0.149)	0.256 (0.002; 0.010)	0.201 (0.017; 0.051)
MCVL	0.096 (0.260; 0.360)	0.178 (0.035; 0.079)	0.104 (0.222; 0.296)
IIC	0.119 (0.161; 0.256)	0.294 (<0.001; <0.005)	0.291 (<0.001; <0.005)

Spearman correlation coefficients (ρ) are presented with nominal *p*-values and Benjamini–Hochberg FDR-adjusted *q*-values. Statistical significance after correction for multiple testing was defined as *q* < 0.05.

## Data Availability

The data used to support the findings of this study are available from the corresponding author upon reasonable request.
